# Wearable Travel Aids for Blind and Partially Sighted People: A Review with a Focus on Design Issues

**DOI:** 10.3390/s22145454

**Published:** 2022-07-21

**Authors:** Marion Hersh

**Affiliations:** Biomedical Engineering, University of Glasgow, Glasgow G12 8QQ, Scotland, UK; marion.hersh@glasgow.ac.uk

**Keywords:** travel aid, blind, design, wearability, features and functions

## Abstract

The ability to travel (independently) is very important for participation in education, work, leisure activities, and all other aspects of modern life. Blind and partially sighted people experience a number of barriers to travel, including inaccessible information and environments, and consequently require support from technology or other people to overcome them. Despite the potential of advanced technologies and the development of electronic travel aids, the long cane and guide dog remains the most commonly used solutions. Wearable technologies are becoming increasingly popular. They have the particular advantage of keeping the hands free, thereby facilitating the use of a long cane, guide dog or another device at the same time. They also have the potential to change the ways in which users interact with the environment. The main contributions of this paper are surveying the current state-of-the-art of travel aids from a design perspective and investigating the following issues: (1) The important design issues in wearable travel aids and the extent to which they are taken into account in different devices; (2) The relationship, if any, between where and how travel aids are worn and their design, features and functions; (3) Limitations of existing devices, gaps in provision and future research directions, particularly with regard to meeting potential users’ needs.

## 1. Introduction

There are about 253 million visually impaired people in the world, 2015 data, with about 39 million blind [1]. A total of 80% of them are 50 or over, and 78% live in low or middle-income countries. Subsequently, the term blind will be used to indicate a person with a significant visual impairment that affects their mobility. However, the term used in the literature will be used to indicate the group(s) of people a particular device is designed for or tested with. Services, facilities and infrastructure are designed for sighted rather than blind people. Consequently, they experience a number of barriers. These barriers affect travel, for instance, through inaccessible information and environments. This impacts the ability of blind people to participate in education, work, leisure activities, and all other aspects of modern life. Therefore, they require support from (assistive) technology or other people to overcome them.

Despite the potential of advanced technologies and the development of electronic travel aids, the long cane and guide dog remains the most commonly used solutions. The long cane is simple, robust, low cost, reliable and requires minimal maintenance. However, it is unable to provide information on distant or high-level obstacles or to support wayfinding and navigation. A survey of 300 blind people found that about 40% experienced head height collisions at least once a year and 15% once a month [2]. The long cane’s visibility and distinctiveness mean that it acts as an indicator that the user is blind, making it easier for them to obtain assistance and other people to take particular care not to bump into them. However, this visibility leads many potential users to avoid its use due to fears of being stigmatised [3]. Guide dogs provide similar guidance to a human guide, but only on known routes. They also have social benefits with regard to companionship and can facilitate interaction with other people. However, they are only suitable for people who like dogs and are able to care for them.

### 1.1. Overview of Travel Aids

There are a number of different ways of classifying travel aids, including their applications, the main technologies used, their form and how they are carried or worn. Classification by their applications and the associated technologies gives three overlapping phases of travel aid development [4]. The first phase focused on obstacle detection devices with additional functionality compared to the long cane. Many of these devices are in the form of a cane, e.g., the laser cane [5], the smart cane [6], the ultracane [7] and the Tom Pouce and Télétact [8]. They use infrared, ultrasonic and/or laser sensors to obtain environmental information and communicate it to users via vibration or non-speech sounds, which are sometimes musical. Some of the more recent devices extract environmental information using camera vision with signal processing of the camera images to identify and sometimes also recognise objects, e.g., [9,10]. This facilitates the addition of object recognition and scene representation functions. Other devices in this category include BBeep [11], which detects people and emits an alert to encourage the detected person to avoid the user. A few aids, e.g., Smart Environment Explorer Stick [12], combine obstacle avoidance and wayfinding/navigation functionalities.

The second phase involved the development of navigation and wayfinding devices using two distinct approaches with overlapping functionality to detect either the user’s location or a point in space [13]. Global navigation satellite systems, most commonly global positioning systems (GPS), locate the user and have point of interest and other functions. GPS systems designed for or which can be used by blind people include Trekker Breeze, Trekker GPS, Navigator and Captain, and software such as Wayfinder on a mobile device [4]. Environmental information beacons locate a point in space using active or passive radio-frequency identification (RFID) tags or infrared transmitters [14], e.g., the Talking Signs system and the Haptic Pointer Interface [4]. They may have additional functions, such as providing information about located facilities or requesting that vehicle doors are opened. More recently, Bluetooth low-energy (BLE) beacons have been used in navigation systems, particularly for large complex indoor environments, e.g., [15,16], but there are other systems that be used both indoors and out, e.g., [17].

BLE systems generally involve apps on smartphones, giving the third or current phase of apps on smart mobile devices and vision sensors linked to smart mobile devices. Thus, there has been a progression from the first two phases, with phase one involving mainly hardware, phase two a combination of hardware and software and phase three purely software, with the hardware provided by an existing mobile device. Many of the apps provide specific contextual information that is relevant to both blind and sighted people, e.g., Find my bus and Find my bus stop. However, the appropriate design for compatibility with audio and tactile output is required to ensure they can be used by blind people. Three-dimensional vision sensors are increasingly being used in navigation, including on mobile devices [14]. Cameras and signal processing are also being used with mobile devices to detect particular types of objects, such as tactile tiles or surfaces [18].

### 1.2. Wearable Devices

The devices discussed so far are portable but generally not also wearable. Wearable devices are becoming increasingly popular and have the advantage of keeping the hands free [19]. This is particularly useful to blind people who may want to use a cane or guide dog or other (travel) device at the same time. There is a growing body of research on wearable devices for blind people, but few devices have gone beyond the prototype stage.

Wearable devices (prototypes) have been developed for a wide range of different applications for blind people in addition to travel. This includes devices to support social interaction, recognise social signals and gestures [20,21], provide information about facial expressions [22], the number of people in their surroundings and their position relative to the user [23] and simulate eye contact [24]. Other applications include reading devices [25,26], reading music notation for people with low vision [27], dancing [28,29], running [30], education [31], colour perception [32], identifying medicines in a cabinet [33] and improving gait [34,35]. The development of devices for deafblind people has focused on tactile communication, using Braille or a deafblind manual alphabet [36,37,38], but also includes other applications such as support for deafblind cat owners [39].

The three previous surveys of wearable assistive devices and wearable travel aids for blind people will now be discussed briefly. Velázquez [40] organises wearable assistive devices by the part of the body or type of garment they are worn on, namely wrist and forearm, tongue, head, vests and belts, and feet. There is some discussion of wearable travel aids, but the focus is on tactile displays to be used on different parts of the body.

Dakopoulos and Bourbakkis [41] consider wearable obstacle avoidance devices rather than travel aids more generally, including navigation and wayfinding devices. They present a number of prototypes and projects and provide what they call ‘maturity’ analysis based on 14 criteria divided into ‘user’ needs and ‘engineer’s perspective’. This includes real-time/fast response, reliability, low cost, ease of learning and use, simplicity, performance, availability and portability (lightweight and small size). However, some of particularly the engineering criteria do not seem appropriate. For instance, wireless connectivity is not relevant to all devices and, unless appropriately managed, can lead to privacy and security risks. Possibly unsurprisingly, none of the systems evaluated has all the features. Users were not confident about the reliability, robustness and performance of any of the systems. This is an area that could benefit from further research. The consideration of only obstacle avoidance systems and the possibility that users did not consider them to have overall benefits compared to the long cane could have been a factor. The authors note the importance of devices that are useful long term rather than having all possible functionalities.

Tapu et al. [42] consider assistive devices, with some portable rather than wearable and many, though by no means all, supporting travel. They divide electronic travel aids into active/sensorial network systems and passive/video camera systems and then further divide these two categories by the type of sensor and type of video camera, respectively. However, this classification does not take account of different types of device functionality and, in particular, the important distinction between obstacle avoidance and navigation/wayfinding devices. There is some overlap between their seven evaluation criteria and those of [41] with regard to real-time use, ease of learning, robustness (to scene dynamics and lighting conditions) and portability. However, their other conditions are specific to object detection and not relevant to other types of travel aid. They also focus on camera vision systems and pay less attention to ultrasonic (and infrared) ones. Consequently, the main focus is head (and body)-worn devices, and no foot-worn ones are included.

Thus, existing surveys of travel devices for blind people are useful but have a number of limitations. This includes a focus on obstacle avoidance systems camera vision technologies and limited attention to other applications, and technologies. A particular limitation is the lack of discussion of wearability and whether and, if so, how this makes a difference to the design.

### 1.3. Paper Contribution

This paper intends to fill some of these gaps and, in particular, to review the literature from a design and wearability perspective. It will do this in the framework of the following three research questions:

1. Identifying the important design issues in wearable travel aids, the extent to which they are taken into account in different devices and any gaps.
2. The relationship, if any, between where and how travel aids are worn and their design, features and functions.
3. Identifying gaps in provision, particularly with regard to meeting potential users’ needs.

Table 1 provides a comparative view of the contributions and other features of the three survey papers and this paper. This shows that this paper has a quite distinct additional contribution compared to the three earlier survey papers.

The papers surveyed have been obtained largely from Google Scholar and the survey paper references. The aim was to cover the diversity of the field with regard to technologies used, applications and how and where devices are worn rather than include all published papers. Search terms included ‘wearable’ different parts of the body that devices could be worn on and various travel aid related terms.

The remainder of the paper is organised as follows. Section 2, Section 3, Section 4 and Section 5 present the wearable devices surveyed, organised by the part of the body they are worn on. Section 6 answers the three research questions presented above, and Section 7 presents brief conclusions. Where devices have components attached to more than one part of the body, e.g., head and waist, they are classified by the authors’ description, if provided, e.g., wrist-worn and the position of the main sensor(s) used to obtain information otherwise.

## 2. Head-Mounted Devices

An overview of the head-mounted devices discussed in this paper is presented in Table 2 at the end of the section. Head-mounted devices are clearly visible and can draw unwelcome attention due to the stigma associated with assistive technology [43]. This can be reduced to some extent by incorporating the device into spectacles, which are relatively common, though there is still some negativity associated with them. Their head-based location makes the appearance of these devices particularly important, as they contribute to the image users present of ethemselves. User acceptance and use may be low if they are unattractive, obtrusive or convey an image counter to the one the user wants to present of themselves.

### 2.1. Sensors Used for Particular Applications

#### 2.1.1. Obstacle Avoidance and Environment Description Devices Using Camera Vision Sensors

Many head-mounted devices use camera vision. Head-mounted cameras avoid many of the difficulties of focusing the camera commonly experienced by blind people and provide a similarly field of view (though the information will need to be presented in tactile or audio format) to that of a sighted person, particularly if the camera is spectacle mounted. Most of the cameras used are mini or micro of varying types, but advances in technology mean that they are able to produce high-quality, high-resolution images. Sometimes cameras are combined with other sensors or GPS. When two cameras are used, the distance to an object can be obtained by triangulation of equivalent points in the separate views from the two cameras if equivalent points can be matched up, for instance, by using a laser pointer to find the two camera images of the same point [44]. Adding an inertial sensor gives gravity-referenced data that can be related to the user’s body [45] to facilitate giving travel directions related to the user.

**Figure 1:**
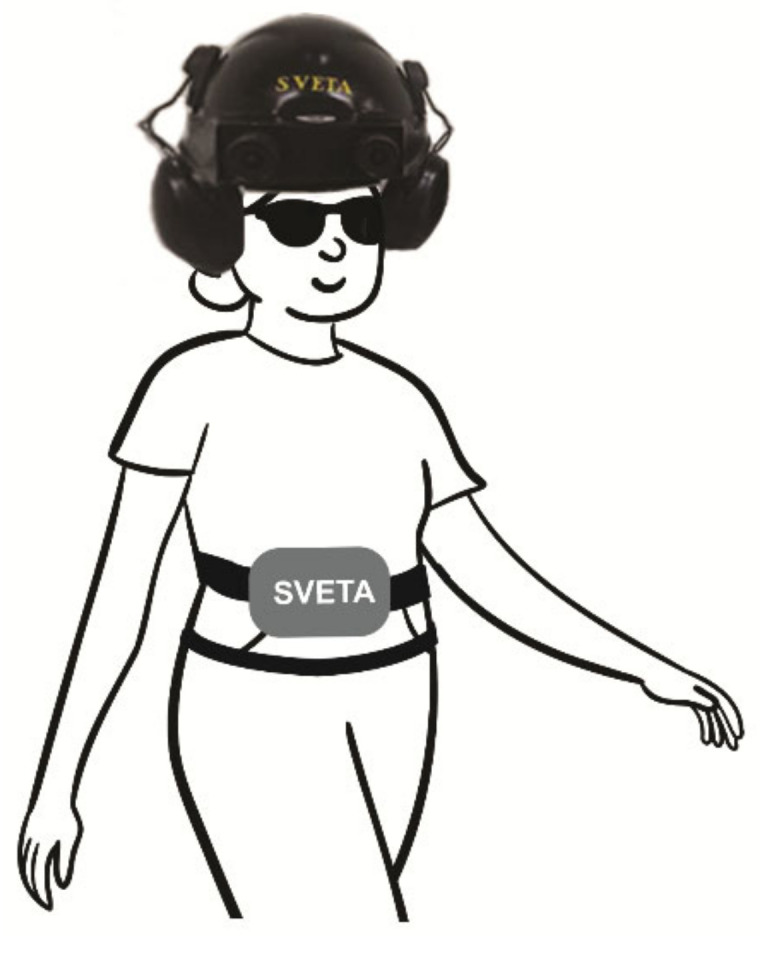
SVETA prototype and blind person wearing SVETA [46].

Six different obstacle detection/avoidance devices with one or two mini cameras mounted on glasses or slightly above the eyes will now be presented. They potentially allow the user to explore the environment by moving their head, similar to a sighted person. The SVETA aid [46] consists of stereo cameras worn slightly above the eyes, earphones and a compact computing device in a waist-worn pouch (see Figure 1). The Intelligent Glasses have two mini cameras mounted on spectacles [45]. The vOICe uses a digital television camera attached to spectacles and connected to a special purpose portable computer [41,47]. Its software has now been loaded onto a mobile phone, making the phone camera available to the device. The camera images are processed without filtering to avoid removing important information.

One of the other three devices uses a compact 3D camera mounted on spectacles and tilted about 45° down and an embedded PC that detects objects in the camera image [48]. Another has two micro-cameras on sunglasses analogously to binocular vision to obtain the disparity between the two images [44]. A laser pointer is used to support the identification of the two camera images at the same point. The third has sunglasses, and a stereo RGB-D camera with processing for deep learning and obstacle avoidance currently carried out on a laptop but intended to be transferred to chips [49]. Training datasets for navigation were collected by a sighted person using the system and used to classify camera images across the categories of go left, right and straight ahead.

The Headlock system [50] is designed to support moving across open areas, which are a particular problem for blind people due to the lack of landmarks. It uses the camera from an optical head-mounted display provided by Google Glass to detect relatively distant landmarks, for instance, doors at up to 12 m, as the user moves their head horizontally to scan the area. In the guidance mode, it detects veering by tracking the landmark position relative to the camera’s field of view and gives the user feedback on the error direction and magnitude to enable them to correct their position.

Other systems aim to provide audio or tactile representations of the local environment. For instance, Sound of Vision uses a stereo RGB camera with a configurable baseline and depth of field camera for outdoor and indoor/low-light image capture, respectively, and an inertial measurement unit (IMU) for tracking head/camera orientation [51]. The cameras and IMU are connected to the central processing unit via a USB 3.0 hub and are placed in a rigid structure that can be connected to headwear. In outdoor environments, a global 3D model is constructed using camera motion estimation and state-of-the-art disparity computation algorithms. Indoors, a point cloud is obtained from the depth map and the camera’s intrinsic parameters. Another system uses two dynamic vision sensors attached to spectacles to obtain visual information [52]. Temporal resolution is increased to microseconds by encoding as a stream of single pixel events compared to the millisecond resolution for frame-based systems. Downsampling is used to reduce the number of events sent to the user to a more realistic number of about 100 per second. The components are all low power, so they should have long battery life.

#### 2.1.2. Navigation Systems That Include Cameras

**Figure 2:**
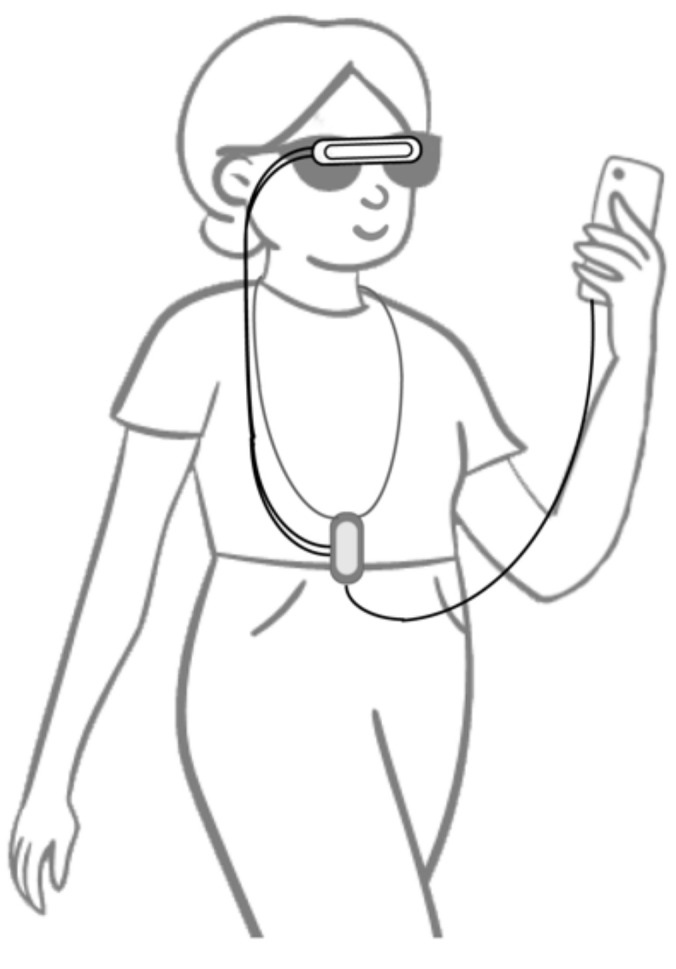
Object detection, recognition and navigation system [53].

Several combined object detection and navigation systems use cameras, often together with GPS. Some of them can be used both indoors and outdoors, and some only either indoors or outdoors. An indoor and outdoor object detection and recognition and navigation system uses an RGB-D camera and inertial measurement unit on spectacles and a smartphone (see Figure 2) [53]. The navigation system uses GPS and VSLAM (visual simultaneous location and mapping) to determine the user location outdoors and indoors, respectively. The guidance modes detect veering by tracking the landmark position relative to the camera’s field of view and give the user feedback on the error direction and size to enable them to correct their position.

An indoor navigation system uses an RGB-D camera on spectacles with an inertial measurement unit sensor on top of the camera for initialisation [54]. The potentially high noise in depth values, particularly from distant objects of interest, is reduced by a simple filtering algorithm using 2D image processing. The navigation algorithm constructs a 3D map of the environment, analyses its traversability and generates a safe and efficient path. The system is currently run on a laptop in a backpack, making it unnecessarily cumbersome. The Tyflos system has two mini stereo cameras attached to sunglasses to create a depth map of the environment [41,55]. Processing is carried out to reduce the map resolution while retaining important information, such as safe paths and objects of interest, and give a 2D representation of the 3D space.

The disadvantages of GPS and camera-based navigation systems include the insufficient precision of GPS and the use of estimates of user position and camera movement rather than exact values. There has been some investigation of potential solutions to these problems. For instance, NAVIG aims to improve user position estimates by using visual landmarks with precisely known positions from the GIS to update the GPS estimates [56,57]. These visual landmarks are obtained from two stereo cameras on a helmet. The GPS and vision estimates are combined using a Bayesian framework with the coordinates of various features from the global information system (GIS) used to remove incoherent user positions. The images are processed by the SpikeNet recognition system to locate visually distinct features, such as shops, buildings and road signs. Computational costs are reduced, and accuracy is increased by running this algorithm on only one camera image and using matching to determine the object coordinates for the second image. Navig is one of the few devices where a participatory design approach involving visually impaired people was used.

A head-mounted stereo camera system aims to support navigation by robust estimation of the camera motion in highly dynamic environments [58]. This is performed by using the global motion property of the ground plane and decomposing the camera motion into motion of the ground plane and motion on this plane. The approach has been demonstrated using image sequences captured by an off-the-shelf-wearable stereo camera with IMU on plastic sunglasses. The system is lightweight and unobtrusive but uses two poor-quality image sensors with a very short baseline.

#### 2.1.3. Devices with Ultrasonic Sensors for Obstacle Detection

**Figure 3:**
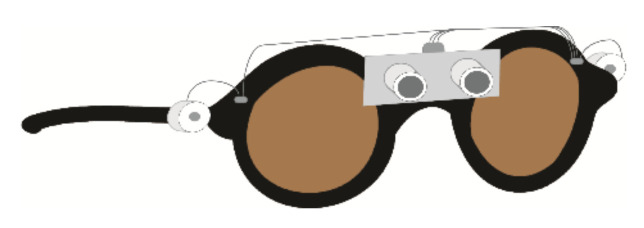
Ultrasonic smart glasses [59].

Other devices use ultrasonic sensors, which are sometimes mounted on spectacles, for instance [59] (see Figure 3). The components are connected to the central unit by single-strand copper wires, which may not be very robust. EyeMate uses ultrasonic range finders mounted on spectacles to detect obstacles to the left and right and on a finger for obstacles on the ground [60]. It also uses GPS to track the user’s position when available, but not for navigation and network provider information otherwise. Another obstacle avoidance device uses five ultrasonic sensors triggered in turn by a raspberry pi 2 working in a continuous loop to detect obstacles [61]. The presence of an obstacle is determined by the minimum recorded distance being less than a threshold value.

A further ultrasonic device uses sonars mounted at the sides of spectacles to detect overhanging obstacles at a distance of up to 6 m and an angle of up to 15° above the head [62]. The analogue distance values are converted to digital values in the controller. The authors suggest using ultrasonic and temperature sensors on a cane on wheels to detect ground-level obstacles and temperature changes, e.g., fire. The cane-mounted sensors are proposed as an alternative rather than to complement the spectacles-mounted ones. The usefulness of the temperature change indicator is unclear, as users would probably smell, hear and feel the heat of a fire at a distance sufficient to give a temperature change.

### 2.2. User Interface and End-User Testing

#### 2.2.1. Devices with Audio User Interfaces

Head-mounted devices most commonly use audio displays, though some devices use tactile displays and others a combination. However, most of the audio displays seem to have been designed without awareness of the importance of not blocking environmental sounds, for instance, through the use of bone conduction earphones. Devices with speech output will be considered first. NAVIG’s voice interface uses Dragon Naturally Speaking and allows users to request a destination, including a room if the building map is embedded or the object known to the system [57]. A system of 3D binaural spatial information over bone conduction headphones is being developed with bone conduction used to avoid masking environmental sounds. An ultrasonic obstacle avoidance device [61] uses speech over Bluetooth headphones to inform the user whether there is an obstacle within the threshold radius and its position (front, slight left or right, hard left or right) and a ‘clear’ message to indicate no obstacles within the threshold distance. The process is repeated continuously, and all detected obstacles reported.

A sunglasses-based device used with a smartphone has voice output over an earpiece with volume decreasing with the distance from the user and preceded by a ding sound [49]. The system also provides go left and right speech instructions to avoid obstacles. However, the need to stop and touch the phone touchscreen in order to receive object information for a particular area is a potential disadvantage, which could reduce its usefulness. Eyemate users are informed of obstacle locations in Bengali or English, and users can dial a pre-saved number to get assistance by pressing a headset button [60]. This is the only device reported that mentions the language used.

A combination of object detection and indoor and outdoor navigation system uses speech over earphones and a beeping sound to give navigation information [53]. Users can move between the three modes by tapping the phone screen once or twice. The different modes allow users to input navigation commands and ask about their location or the surroundings; receive walking instructions and alerts to obstacles and arrival; and obtain information about object category, location and orientation. Message prioritisation is used to reduce cognitive load. Initial tests with 20 blind and partially sighted people obtained shorter walking times and fewer collisions with the system than using a long cane and navigation instructions. The object recognition system could be used, for instance, to determine whether an object blocking the path could be moved or needed to be walked around.

Other devices use non-speech sounds. For instance, an ultrasonic object detection aid uses a buzzer with faster beeping indicating closer obstacles [59]. Devices which provide environmental information rather than just obstacle locations often use sonification schemes. The SVETA aid [46] uses stereo musical sounds, with the sound amplitude providing distance information, the frequency the vertical orientation, and the left and right channels the horizontal orientation. Another environment description system [52] uses virtual spatial sonification to produce sounds that appear to be at the location of events.

A camera-based object detection aid uses sound output over headphones to provide auditory localisation cues that enable users to locate the distance and direction of objects [44]. The user is able to choose the sonification approach by moving the laser pointer in a particular way. The vOICe sends unfiltered camera images to a one-to-one image to sound mapping. The user receives the sound over headphones [41,47]. Promising results have been obtained from end-user tests after extensive training. However, the potential difficulties in understanding this sound scheme should be noted.

The spatial location of sound and using the different sound features to convey different types of information, for instance as a result of the exploration strategies used can have benefits, as found with vOICe. However, users may require an extensive period to learn to use such systems effectively. There are also issues of whether the majority or only some potential users will be able to learn to use devices with complex sound systems effectively and whether the additional concentration required is likely to distract attention from important environmental sounds.

#### 2.2.2. Devices with Tactile Displays and Combined Audio and Tactile Feedback

**Figure 4:**
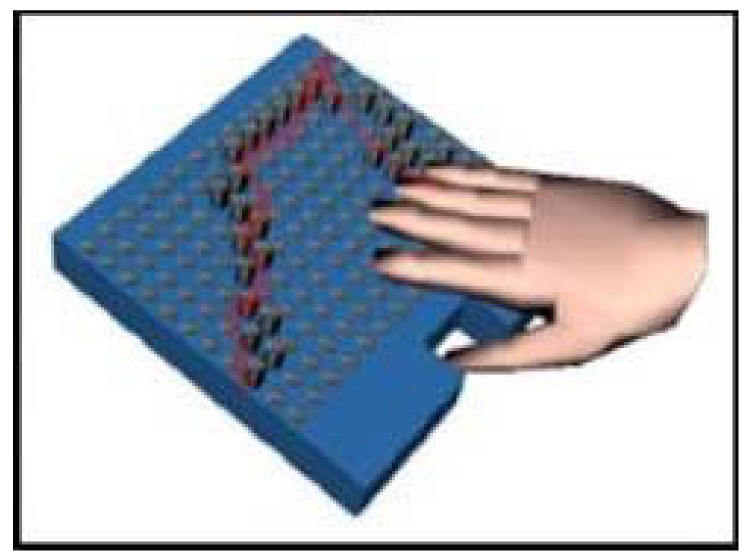
Intelligent glasses and its tactile display [45].

Several devices use tactile displays. Most of them are passive and provide information to users by vibration. Active displays can be explored by users, potentially allowing access to more information and giving users control, but at the risk of them missing important information. It may also be difficult for them to carry out exploration while walking. The intelligent glasses [45] are the only device surveyed with an active display (see Figure 4). Users can freely explore a map of obstacle locations on a tactile display using miniature actuators of shape memory alloy.

Tyflos uses a 4 × 4 array of vibrators on an elastic vest on the user’s abdomen with varying vibration frequencies controlled by a microprocessor and portable computer [41,55]. It provides information about ground and head height obstacles, with the direction represented by the vibrator position and the distance by the vibration levels. However, the benefits of a 3D representation could be offset by its complexity and difficulties in learning to understand it. A camera obstacle avoidance system has vibrating motors in a vest on the shoulders and waist [63]. The motor closest to the nearest continuous free path vibrates to indicate the direction to take, but the authors intend to develop a more complex route planning algorithm, for instance, to avoid trap situations. An indoor navigation system also has tactile actuators on a vest, which indicate right and left turns, continue and stop and scan [54]. The user can communicate with the system using a smartphone. Initial experiments with small numbers of blindfolded participants gave reasonable results, but considerable testing against other aids will be required.

Several tongue-stimulating displays have been developed, but the focus has generally been the display rather than the development of a complete travel aid. A device which obtains environmental images from a spectacles-worn camera transmits the processed signal wirelessly to a 6 × 6 circular electrotactile display worn in an orthodontic retainer [64]. The final version is intended to include all components other than the camera in the retainer. Tongue displays use the tactile sensitivity of the oral cavity, which is similar to that of the hands. However, there are a number of potential disadvantages, making it likely that user acceptance will be low. They include retainer appearance, possible negative effects on speech and the need for careful cleaning.

Some devices use both audio and tactile information. For instance, a camera-based object detection aid [48] provides information about obstacles using three vibrotactile actuators worn on armbands on each arm and a band on, for instance, the back of the neck and audio messages over a bone conduction headset to give the average distance to central objects. An ultrasonic device uses a combination of audio messages and vibrating motors attached to the fingers to indicate distant, moderately distant and close obstacles [62]. In the Sound of Vision system, users receive audio and haptic information on the size, type, location and elevation of objects. They choose the number of objects to be encoded in indoor environments and how they should be chosen to avoid disorientation when there are a lot of objects [51].

There are also devices with visual displays that transform information to present it in a format that is more accessible to particular groups of partially sighted users. For instance, augmented reality digital spectacles [65] use digital video reprocessing algorithms involving image remapping and data on the user’s visual field to expand the functional visual field of people with reduced visual fields. The algorithm might need to be separately calibrated for each user. The approach can support mobility by providing visual access to information about objects in the peripheral visual field that would not otherwise be available.

**Table 2:** Device features and testing for head-mounted devices.

Reference	Where Worn	Functions	Sensors	Feedback	Testing
Agarwal et al., 2017 [59]	Head	Detects obstacles in front at 300–3000 m	2 sonars on glasses	Beeping on buzzer	No end-user testing
Bai et al., 2019 [53]	Head	Indoor and outdoor object detection, recognition and navigation	RGB-D camera and IMU on glasses, GPS	Speech over earphones and beeping sound	20 blind people
Balakrishnan et al., 2007 [46]	Head and waist	Object identification and obstacle avoidance	Stereo cameras in helmet above eyes	Stereo musical sounds over earphones	Blind and sighted tested 3 sound systems
Bharathi et al., 2012 [62]	Head and fingers	Detects above-head or ground obstacles	Sonar at side of glasses or cane	Beep, 3 vibrating motors on fingers	No end-user testing
Brilhault et al., 2011 [56] Katz et al., 2012 [57]	Head	Improves user localisation	2 stereo cameras, GPS, IMU	Developing 3D sound localisation system over bone conduction headphones	Blind and sighted tested 3 sound systems
Caraiman et al., 2017 [51]	Head	3D audio/tactile representation of environment	Stereo RGB-D and depth of field cameras, IMU	Audio and haptic object information	19 visually impaired, modelled indoor area
Dakopoulos, 2009 [55]	Head and abdomen	Environmental representation and safe navigation	2 mini stereo cameras attached to sunglasses	4 × 4 vibrator array in vest on abdomen	10 sighted, 2 visually impaired
Everding et al., 2016 [52]	Head	Audio information about environment	Two dynamic vision sensors	Virtual spatial sonification	11 unspecified, user tests of functioning, not as travel aid
Fiannaca et al., 2014 [50]	Head	Moving across open areas	Google glass camera	Speech, 3 high/low pitch beeps	5 blind people navigation to door
Fusiello et al., 2002 [44]	Head	Sound map of visual space	2 micro cameras on sunglasses	Sonification over headphones	Unspecified, tests of sonification scheme
Laubhan et al., 2016 [61]	Head	Obstacle detection	5 sonars	Speech output over headphones	3 tests with 2 users
Lee and Medioni, 2014 [54]	Head and chest	Indoor navigation	RGB-D camera on glasses	Tactile actuators on vest	4 blindfolded sighted, cluttered space
Leung et al., 2014 [58]	Head	Robust estimation of camera motion	Stereo camera	Not stated	No end-user testing
Lin et al., 2019 [49]	Head	Object identification and obstacle avoidance	Stereo RGB-D camera on sunglasses	Speech over earpiece	20 blind people. Compared to long cane
Mattoccia and Macri, 2014 [48]	Head	Obstacle detection	Stereo camera on glasses	3 vibrating motors, bone conduction audio	1 blind, 3 blindfolded sighted outside and inside
Meijer, 1992 [47] Dakopoulos and Bourbakis, 2009 [41]	Head	Environmental representation	Digital TV camera on glasses	Sonification over headphones	Unspecified, good results after extensive training
Pradeep et al., 2010 [63]	Head, shoulders and waist	Obstacle avoidance	Stereo camera	Vibrating motors on shoulders and waist	16 blindfolded sighted
Sayed et al., 2020 [65]	Head	Presenting information from peripheral visual field	Miniature camera and eye tracking system	Peripheral visual field images presented in central part of glasses.	21 people with reduced visual field
Tanveer et al., 2015 [60]	Head and finger	Obstacle detection, user tracking	Sonars on glasses and finger ring, GPS	Bengali or English obstacle locations	No end-user testing
Velazquez et al., 2006 [40]	Head and waist	Tactile map of visual space and possible navigation paths	2 stereo cameras on glasses, inertial sensor	Waist worn array of tactors	20 sighted, tactile tasks, virtual environments

## 3. Body-Worn Devices

An overview of the body-worn devices discussed in this paper is presented in Table 3 at the end of the section.

### 3.1. Sensors

#### 3.1.1. Devices with Ultrasonic or Infrared Sensors

Several devices use infrared or ultrasonic sensors on a waist belt for obstacle detection, with sonars more commonly used. ALVU uses seven infrared sensors worn on the front of a waist belt to detect obstacles in a cone with an angle of 70° about the forward direction [66]. The intelligent belt uses four infrared sensors interfaced with a microcontroller circuit to detect obstacles at the front, left, right and back [67].

**Figure 5:**
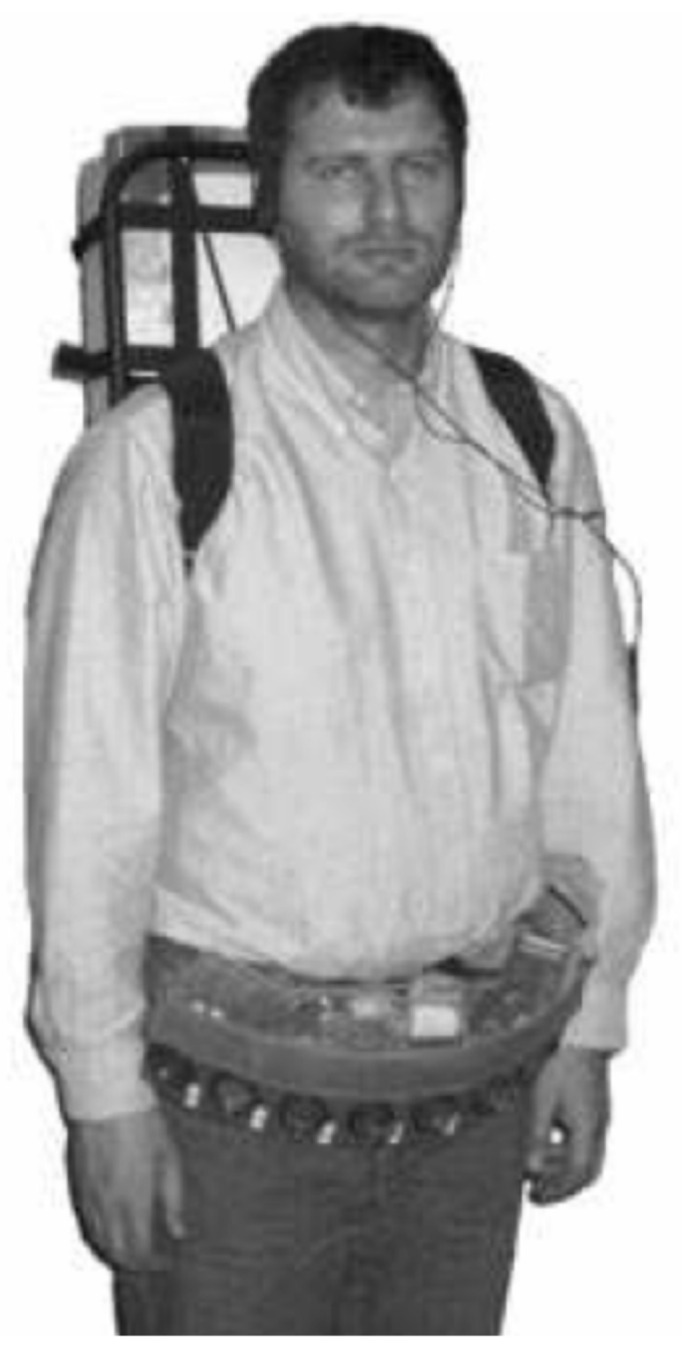
NavBelt [68].

The NavBelt [68] uses eight ultrasonic sensors on a waist belt (see Figure 5) to detect obstacles with total coverage of 120°. Better results have been obtained from setting the sonar range to 3 m rather than 2 m. The portable computer in a backpack could presumably be replaced by, for instance, software on a smartphone to make the device smaller and lighter. Another device uses ultrasonic sensors on a waist belt to detect obstacles to the right, left and in front and is controlled by an Arduino nano microcontroller [69]. A Bluetooth link enables the user to control the system using their mobile phone.

Another belt-worn device, the ActiveBelt, uses a belt-worn GPS and a direction sensor comprising geomagnetic and acceleration sensors to detect the user’s location and orientation [70]. A microcontroller is used to control the system sensors and vibrators. It would need to be used together with a long cane. This device has been included as it seems to be suitable for blind people, though presumably designed for the general population. The idea of devices that are suitable as travel aids for blind people and also of interest to sighted people is an interesting one.

Other devices have ultrasonic sensors on bands or straps on different parts of the body. The wearable virtual white cane network uses ultrasonic sensors worn on bands on the waist, wrists and one ankle to detect obstacles in front, to the left and right of the user and at a low level, such as stairs [71]. The sensor and battery are on the lid of a small box, and the other components are on the case or inside the box. Each component is controlled separately by its own microprocessor. Another ultrasonic obstacle detection device uses commercially available components, including a potentiometer, microcontroller and Nokia coin vibrator motor enclosed in a custom acrylic package worn on a neck strap [72]. The user can calibrate the detection range.

Some devices involve small units containing a sensor and vibrator that can be worn on different parts of the body. For instance, Uasisi consists of tiny vibrating modules which use sonar echolocation to detect obstacles [73]. They are constructed from off-the-shelf components and linked together, and can be embedded in wearable items such as bracelets, hats and belts. They can be linked to a smart environment to provide additional information, such as points of interest. Vista wearable uses small enclosures containing an infrared range sensor, vibrator, battery and microcontroller to detect and provide direct feedback on nearby obstacles and walls [74]. They can be clipped to armbands, pockets and clothing. A Bluetooth low-energy wireless interface enables multiple units to be controlled through an existing single device, such as a smartphone, and allows the sensor and vibration units to be separated into two pods, which could be worn over and under a coat in the winter. Watch your head uses two ultrasonic transducers, which can be worn on a shirt pocket or in a brooch, to detect head height obstacles that cannot be detected by the long cane and could be misjudged by a guide dog [75]. It should be used together with a long cane or guide dog to provide the additional functionality required for safe mobility. It uses state-of-the-art signal processing and off-the-shelf hardware components to reduce costs. Average power consumption is expected to be less than 50 mW, giving 90 h of continuous use from a 500 mAh 9V battery.

Ultrasonic sensors, together with GPS, are used in combined obstacle avoidance and navigation systems. For instance, a device with sonars on a waist belt can detect obstacles within a metre to the left, front and right and combines them with smart spectacles and GPS [76]. The device range is relatively short, particularly for people with a long stride, and could, therefore, give users insufficient time to avoid detected obstacles. GPS and Google Maps are used to locate the user, and text messages of their location can be sent to an assistant who asks for it via text message. This gives rise to the risk of unauthorised people accessing the user’s location. There also seems to be a tacit assumption that assistants should be able to track blind people’s locations rather than the location being available for the blind person to communicate if they require assistance.

#### 3.1.2. Devices with Cameras and Ultrasonic Sensors

Some obstacle avoidance devices have both ultrasonic sensors and cameras. A combined obstacle avoidance and person detection system uses belt-mounted ultrasonic sensors for obstacle detection and a head-mounted USB webcam for person detection [10]. Person detection is based on face detection when a face is visible or the detection of cloth next to skin otherwise. A sonar/camera device for detecting and recognising static and dynamic obstacles combines information from four ultrasonic sensors and a smartphone video camera, both worn on a waist belt [77]. A filtering strategy is used to reduce the number of points of interest from the camera to a manageable number. Obstacles are also detected using the sonar sensors arranged horizontally with some overlap to maximise coverage. Tests indicate that the combination of sensors leads to improved obstacle detection, but the authors do not discuss or explain how this is achieved. Obstacle recognition involves a training image database organised into vehicles, bicycles, people and obstructions.

Several devices, with obstacle detection and classification, navigation and/or combined obstacle avoidance and navigation, use a camera and may be used together with a long cane as a primary obstacle avoidance device. A smartphone obstacle detection and classification system uses the phone camera, which is harnessed to the chest [78]. Points of interest are selected from the image grid using a grid sampling strategy, the motion of these points is tracked, and the motion of the camera/background is estimated. Object recognition involves a modified version of the histogram of oriented gradients descriptor, development of visual descriptors, supervised learning and object identification.

ISANA [79] uses an infrared depth camera running on a Tango android mobile tablet and 2D depth projection to update obstacle positions on the navigation map and detect obstacles in front. It is worn in a holder hanging from the neck and used with a long cane. It draws on the architectural floor plan to obtain a semantic map of the building floor with hallways and some door labels. The map is aligned by the user walking around, using scene recognition and screen tactile input. It is used with obstacle detection to update the 2D grid traversability map, which is used to generate a safe route. Another indoor navigation system consists of a chest-worn high-resolution stereo RGB-D camera and a high-computation capacity embedded processor [80].

A device designed specifically for deafblind people uses a fisheye camera (very wide-angle lens) just under the neckline of a haptic vest to obtain environmental information, support navigation and locate nearby people [81]. A navigation device intended to be used with the long cane uses a 3D-printed RGB camera to obtain depth information [82]. This is used to predict a safe route, determine flat routes and inform the user of the distance to the safe path and ensure each step is safe. The camera and other components are organised on a small box that has both a long strap to hang from the user’s neck and an elastic belt to go around their waist. The system uses deep learning to obtain depth images, calculate the plane for object detection and determine safe walking routes. It uses convolutional neural networks to learn a large number of routes. Another indoor navigation device uses a wireless inertial sensor system worn on the user’s hip and comprises an accelerometer and one and two-axis gyroscopes [83]. It is used with an app on a smartphone, and the inertial sensor system could be replaced by smartphone sensors. The system uses pedestrian dead reckoning algorithms combined with the planned trajectory to estimate the user’s position. Sensed turns are compared with map features to reduce errors.

### 3.2. User Interface and End-User Testing

#### 3.2.1. Devices with Tactile Displays

Many body-worn devices, particularly those on a waist belt, communicate information to users through vibration and, consequently, are likely to be suitable for deafblind people. ALVU provides information to the user through feedback motors 8–12 cm apart on a haptic strap on the upper abdomen. This is considerably greater than the minimum spacing required to distinguish two tactile stimuli on the torso [84] and allows an intuitive mapping to sensor positions and, consequently, object directions. Uasisi uses small vibrating motors embedded in wearable items to indicate the presence of a nearby object, with increasing frequency as it gets closer [73]. Vista wearable uses vibrators in small body-worn units to inform the user about nearby objects and walls [74].

**Figure 6:**
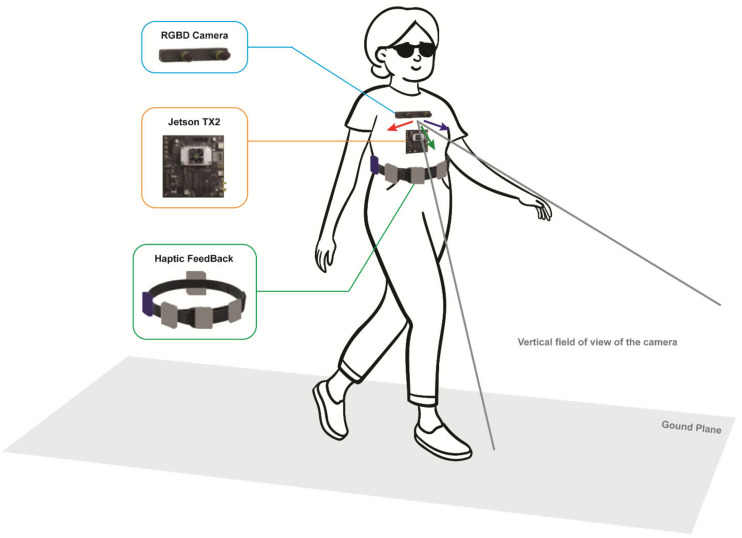
Camera and GPS navigation system with haptic feedback [80].

An indoor navigation system uses four vibrating motors at the front and back of a belt and 30° to the left and right of the front (see Figure 6) to indicate the detection of a target object, the travel direction and that the user should stop and scan for a path [79]. A neck-worn obstacle avoidance device uses a Nokia phone coin vibrator to alert the user to obstacles [72]. Its rechargeable polymer lithium-ion battery is estimated to have 288 mW power consumption and eight-hour battery life. This is probably greater in practice, as the vibrator, which is responsible for most of the power consumption, is not in constant use.

A navigation device for deafblind people has five vibration motors around the waist area of the haptic vest and two vibration motors near each of the collarbones [81]. The vibration of one of the waist motors indicates the walking direction. The presence and distance of a person or object are also indicated by the vibration of these motors, with low frequencies indicating getting too close, medium frequencies an optimal distance from a person and high frequencies too far apart. Tapping sensations on the back and front shoulder blades are used to indicate start walking/go and stop walking/stop, respectively. Preliminary tests with five deafblind people found that they could follow directional cues and complete the pre-defined route, and led to a suggestion of using stronger haptic signals on the shoulders.

The ActiveBelt has eight vibrators attached inside a belt, but subsequent user trials have indicated that four would probably be sufficient [70]. The original fixed-length version was modified with elastic rubber parts to fit it to the user’s waist and to avoid sensor positions moving away from designated directions as the belt was tightened. However, the use of leather to attach the vibrators for the fixed sections is unsuitable for the increasing number of vegans, and a number of people are allergic to rubber. The user can register their destination with a host PC and be guided to the destination using vibration in the appropriate direction, with vibration intervals reducing as the user approaches the destination. However, this could put users at risk of unauthorised people having access to information about their destinations. The system can also alert users to the locations of information of interest. Insufficient information is provided about the differences between the vibration signals used in navigation and to alert users to points of interest required to avoid users confusing them.

#### 3.2.2. Devices with Audio and Combined Audio and Tactile Displays

A few devices, such as the virtual white cane network (VWCN) [71] and Watch your head [75], provide both vibrotactile and audio feedback. The vibration and sound magnitudes of the VWCN vibrating motors and buzzers are mapped linearly to the obstacle distance and threshold value, which can be set at multiples of 80 cm between 80 and 480 cm. The relatively low current gives 9.5+ h of continuous operation for a 400 mAh battery. Tests with blindfolded sighted users found that using the long cane and device together considerably reduced navigational errors compared to either device on its own. An obstacle detection and navigation device uses a combination of programmed voice instructions to indicate the walking direction and vibration for deafblind users to indicate obstacles [69].

A few devices of different types provide speech output to the user. This has the advantages of allowing more detailed information to be conveyed and not requiring interpretation and the disadvantage of blocking environmental sounds unless used with bone conduction headphones. ISANA [79] uses real-time speech guidance and alerts to inform users of a safe route. A priority mechanism is used to reduce cognitive load. An ultrasonic and GPS device uses speech messages [76]. In addition, the user’s location obtained from the GPS is available to an assistant via a text message sent to the device. The user can press a button to automatically send a text message to an assistant with a link indicating their location on Google Maps if they require assistance. Both these features raise potential privacy and security issues. Enabling users to send a message with their location to another person who might be able to provide useful information if they get lost or otherwise require assistance is clearly a useful option. However, there seems to be a tacit assumption of dependence and that, consequently, another person is entitled to know the user’s location.

Another indoor navigation system uses text-to-speech audio cues for map features, such as upcoming turns and points of interest triggered by approaching them [84]. A combined ultrasonic/camera device uses speech messages to transmit speech alerts of static or dynamic obstacles and is one of the few devices to use (Bluetooth) bone conduction headphones [78]. Messages are prioritised by potential risk, which is indicated by ‘urgent’ or ‘normal’ before the object name. Testing involved 21 visually impaired people in unfamiliar urban environments. Most participants considered the device very useful when used together with the long cane and found it wearable and lightweight.

Other devices use non-speech sounds. The Navbelt uses sounds over small stereophonic headphones to provide three modes of use [68]. Virtual directions are obtained from a binaural feedback system using the phase and amplitude differences of the sound at the two ears. In the guidance mode, single stereophonic tones guide the user around obstacles, with the direction giving the travel direction and higher frequencies indicating lower recommended travel speeds. The image mode uses stereophonic sounds to give a panoramic acoustic image of the environment. In directional guidance mode, the user determines the direction using a joystick (to be replaced by an auditory coding system or speech control device), and the device avoids obstacles. The intelligent belt uses pre-recorded messages over headphones to tell the user which direction to walk in [67]. An RGB system for identifying safe walking routes uses audio information over earphones with the sound amplitude used to indicate when the user should continue walking [82]. The developers of a smartphone obstacle detection and classification system plan to add an advanced alerting system that does not obstruct environmental sounds [78].

**Table 3:** Device features and testing for body-worn devices.

Reference	Where Worn	Functions	Sensors	Feedback	Testing
Diaz et al., 2020 [80]	Chest	Indoor navigation	High-resolution stereo RGB-D camera	4 vibrating motors on belt	2 blindfolded sighted inside
Gao et al., 2015 [71]	Waist, wrists, ankle	Obstacle detection in front, left, right and low	Sonars on waist, wrists and one ankle	Vibrating motors and buzzers	15 blindfolded sighted people
Garcia-Macias et al., 2019 [73]	Different body parts	Indicating nearby objects	Sonar on small wearable items	small vibrating motors in wearable items	No end-user tests of device in use
Gay et al., 2020 [81]	Waist, top of chest and shoulders	Navigation, distance to person or object	Fisheye camera on haptic vest	4 vibrating motors on shoulders, 5 on waist	5 deafblind, complete pre-defined route
Hsieh et al., 2020 [82]	Chest or waist	Detecting indoor objects and safe walking routes	3D-printed RGB camera	Sound over earphones	No end-user testing
Jameson and Manduchi 2010 [75]	Chest	Alert to head height obstacles	2 sonars	Audio or tactile alert	No end-user testing
Li et al., 2016 [79]	Round neck	Indoor navigation and sign reading	Infrared depth camera	Real-time speech guidance and alerts	No end-user testing
Mocanu et al., 2016 [77]	Chest	Obstacle detection and recognition	4 sonars, phone video camera	Speech over bone conduction headphones	21 visually impaired, outdoors
Molina et al., 2015 [74]	Different parts of body	Awareness of surroundings	IR sensor in wearable small enclosure	Vibrators to warn about objects and walls	5 blind, 6 low vision, 10–20 h daily life
Prathipa et al., 2019 [69]	Waist	Obstacle detection and avoidance	Sonar on waist belt	Pre-recorded speech, vibrating motors	No end-user testing
Riehle et al., 2013 [83]	Hip	Navigation with environmental info	Accelerometer and 1 and 2 axis gyroscopes	Speech alerts	8 blind, 8 sighted, shopping centre
Shoval et al., 1998 [68]	Waist	Navigation, acoustic image of environment	8 sonars	Sounds over small stereo headphones	Sighted people, obstacle avoidance
Tapu et al., 2013 [78]	Chest	Obstacle detection and classification	Camera on smartphone	Plans to add—not block environmental sounds	No end-user testing
Tsukada and Yasumura 2004 [70]	Waist	Obtaining directional information	GPS, geomagnetic and acceleration sensors	8 vibrators inside adjustable belt	Sighted people can find direction
Venkateswar and Mehendale, 2012 [67]	Waist	Obstacle detection	4 infrared sensors on belt	Pre-recorded messages over headphones	No end-user testing
Villamizar	Chest	Obstacle detection, calculate range	Ultrasonic	Phone coin vibrator	10 visually impaired determine detection range; 5 unspecified
Yeboah	Waist and head	Obstacle detection	Sonars on waist belt and GPS	Speech messages	Some end-user testing but no details

## 4. Hand and Arm-Worn Systems

An overview of the hand and arm-worn devices discussed in this paper is presented in Table 4 at the end of the section.

### 4.1. Sensors

The fingertip skin is one of the most sensitive areas of the body, but devices worn on the fingertips could possibly be lost or damaged. They could also impede the free movement of the fingers and make it more difficult to use the hands for carrying and other activities. In addition, each fingertip only has a limited surface area, making it unrealistic to attach more than one tactile sensor, though this could be resolved by the use of several fingertips. Consequently, many of the devices in this category are in the form of gloves or wristbands.

Several glove and bracelet devices use ultrasonic sensors, with bracelet devices more common. A smart glove obstacle detection system has ultrasonic sensors with a range of 4 m to detect obstacles and a LilyPad Arduino microcontroller to make it lightweight, inexpensive, wearable and washable [85]. An ultrasonic bracelet has an ultrasonic transceiver with good acoustic and electrical noise resistance on a customised bracelet to detect obstacles in the range of 20 cm to 6 m and calculate their distance [86]. The strength of the echo depends on the angle of the object’s surface facing the receiver. All components can be integrated into the bracelet, but this reduces battery life, or the transmitter worn on the bracelet and the receiver attached to a belt or put in a pocket. The device is able to detect waist to chest level obstacles at front, left and right, and should be used together with a long cane for low-level obstacles.

**Figure 7:**
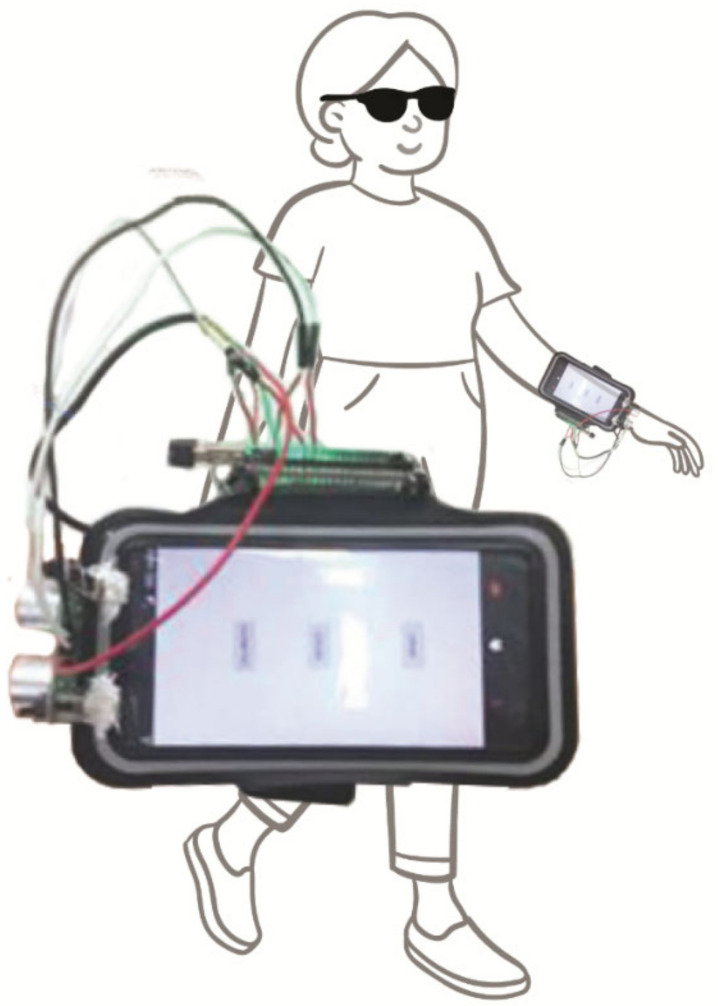
Ultrasonic wrist-worn device [88].

An ultrasonic obstacle detection device, which can be wrist-worn, uses two ultrasonic transducers transmitting identical ultrasonic pulses and controlled by the same input signal [87]. This enables detection over a wider range than a single sonar. Object detection reliability is improved by rotating the two sonars outward to give a vergence angle and increase the delay between the two detected echoes. The system is able to determine which sonar detected the echo first and, consequently, whether it is to the right or left of the user. Another ultrasonic device detects obstacles using data from the ultrasonic sensor and the accelerometer in the linked phone; both mounted on a wristband (see Figure 7) [88]. Both sensors are required to detect ground-level obstacles, and only the sonar to detect above-ground obstacles. System use requires an app to be installed on the phone. Bluetooth is used to transmit data and commands between the phone and the microcontroller connected to the sonar. Tests with five blindfolded sighted people who had received some training in using the device and the long cane showed better performance in terms of obstacle avoidance and speed on indoor paths with obstacles.

There are also wrist-worn and glove-based devices that use cameras, though the camera is generally worn on another part of the body. A wrist-worn obstacle avoidance system uses a Kinect sensor with a viewing angle of 57.5° connected to the lower abdomen [89]. The system calculates the directional angle for the user to walk to avoid the obstacle. The power bank and laptop for processing sensor data are carried in a backpack, making the system rather cumbersome, though the laptop could presumably be replaced by a smaller computing device. A haptic glove uses a stereo camera to obtain a depth map for a distance up to about 10 m, but the simple mapping used is unable to detect curbs, stairs and ground changes [90]. The glove is interfaced with a USB from which it draws power, with a total requirement of less than 150 mA. Testing involved nine visually impaired people and two indoor courses with boxes as obstacles.

Another camera system uses image processing from a chest-worn monochromatic or colour RGB camera (with the other components on gloves) to support users walking or running along lanes or lines [91]. Since most pavements are not marked with lines, the system may be more useful for running on tracks, though the maximum speed of 10 km per hour could be limiting, particularly for fast runners. However, it could have a role in indicating the boundaries of same-level cycle and walking tracks without tactile indications and purely visual walking or location markings, for instance, in conference venues.

### 4.2. User Interface and End-User Testing

**Figure 8:**
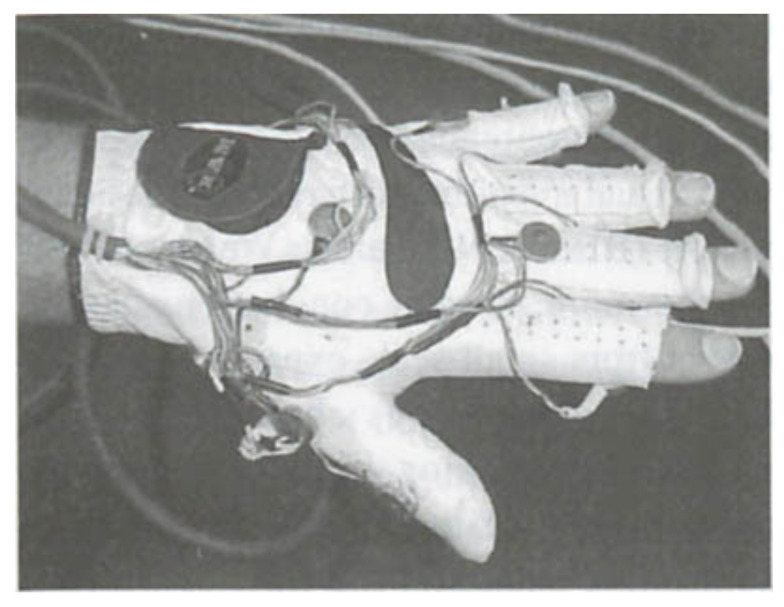
Tactile glove [90].

The glove and bracelet-worn systems generally use tactile displays to provide information to users. An ultrasonic smart glove alerts users with a vibrating motor when obstacles are within 0; 762 m [85]. This distance could be modified and should probably be increased, as it is, for instance, considerably shorter than the long cane detection distance. Tests indicate that device performance and obstacle detection depend on the height at which it is held. Though potentially allowing for scanning for obstacles at different heights, this could complicate its use and lead to ground level or other obstacles being missed. A glove with a camera system has 14 tiny vibrating mechanical pager motions on the fingers and other locations (see Figure 8). The motor locations have been chosen to enable the vibration of the separate motors to be distinguished when several motors vibrate [90]. The glove is powered by a universal serial bus connector, and the motors draw less than 150 mA in total. A glove to support running and walking on lines or lanes uses vibrating motors on the gloves to communicate the direction and whether the user should maintain, increase or reduce their speed [92].

An ultrasonic device with two transducers with identical pulses uses pager motors mounted on both sides of the sonar and which could be worn on the wrists [88]. They provide right/left directional and distance information, with reduced vibration speed indicating greater distance. A wrist-worn obstacle avoidance system uses seven solenoids to provide haptic feedback by tapping the skin [89]. The left and right solenoids indicate the side, and the others indicate the obstacle angle in a binary number system. However, this seems to be excessively complicated and difficult to interpret and could lead to errors and misunderstandings. Wristband size and solenoid spacing are based on one of the authors’ wrists but may not be suitable for people with considerably smaller or larger wrists.

Some of the wrist-worn devices provide both tactile and audio output or a choice between them. An ultrasonic bracelet uses variable frequency vibration to indicate obstacle distance, with buzzer beeps for nearby obstacles [86]. A wrist-worn device linked to a smartphone gives users a choice of audio or tactile alerts [88]. It requires user calibration on first use by the user moving their arm up and down slowly. Further arm movements can be used to activate the detection of ground-level and above-ground-level obstacles, set the detection range between 20 cm and 5 m, and determine whether obstacle alerts are audio or tactile.

Other applications for which wristband and glove devices have been used include route learning and traffic light indicators. For instance, a tactile wristband has been used to support route learning from tactile maps by programming the vibration patterns of a vibration motor using an Arduino Bluetooth board [92]. Frequency, duration and stimuli can be controlled. The direction to follow at each intersection when the user moves their finger over the map is indicated by the vibration pattern.

**Table 4:** Device features and testing for hand and arm-worn devices.

Reference	Where Worn	Functions	Sensors	Feedback	Testing
Alayon et al., 2020 [89]	Wrist, lower abdomen, back	Obstacle avoidance	Kinect sensor	7 solenoids to indicate side and obstacle angle	Limited information on end-user testing
Bhatlawande et al., 2013 [86]	Wrist, possibly waist or pocket	Obstacle detection and avoidance	Ultrasonic sensor	Variable frequency vibration, buzzer beeps	2 blindfolded sighted people on short course
Brock et al., 2014 [92]	Wrist	Route learning from tactile map	Moving finger over map	Vibrating motor	6 blindfolded sighted
Huang et al., 2017 [93]	Hand	Phases of traffic lights	Not stated	Vibrator on glove	Blind performed better than blindfolded sighted
Khampachua et al., 2016 [88]	Wrist	Obstacle detection and avoidance	Ultrasonic sensor and phone accelerometer	Choice of audio and tactile alerts	Blindfolded sighted people
Kuc, 2002 [87]	Possibly wrists	Obstacle detection and avoidance	2 sonars—identical pulses, 1 control input	2 pager motors next to sonars	2 blind people
Linn et al., 2017 [85]	Hand	Obstacle avoidance	Sonars with 4 m range	Vibrating motor alerts to obstacles	2 blind participants in controlled environment
Mancini et al., 2018 [91]	Hand and chest	Following lines when walking or running	Mono or colour RGB camera on chest	Vibrating motors on gloves	No end-user testing
Zelek et al., 2003 [90]	Hand	Obstacle avoidance with range of up to 10 m	Stereo camera	14 tiny vibrating pager motors on glove	9 blind participants on 2 obstacle courses

A glove-worn traffic light indicator uses three different vibration patterns to inform users of the traffic light phase via a vibrator on the back of the glove [94]. Other components are worn on the arm and attached to the glove. The current prototype is fairly conspicuous, and design improvements will be required to make it less obtrusive or more attractive. While the preferred option should be audio and tactile indicators on all traffic lights, this is not yet the case, with not all traffic lights having an audio signal and few having a tactile one. The authors do not state how the device determines the traffic light phase. A study of 18 visually impaired and 18 blindfolded sighted people found that tactile traffic light recognition was greater for the visually impaired than blindfolded sighted participants, particularly outdoors.

## 5. Foot-Worn Devices

A number of different shoe-based travel aids have been developed. An overview of the hand foot-worn devices discussed in this paper is presented in Table 5 at the end of the section.

Incorporating devices in shoes makes them inconspicuous, which is generally a desirable feature of assistive devices [5]. The surface area of the foot is generally sufficiently large to support several vibrators or other factors. The soles of the feet follow the hands in sensitivity to vibration, and the big toe follows the face and fingers in sensitivity to point localisation, e.g., [94]. However, the feet are not particularly sensitive to pressure. Walking comfort should also be a priority of device design. In particular, any travel aid components added to shoes should not cause blisters or other irritation when walking, make gait awkward or reduce speed.

A common design is based on an inexpensive foam insole into which vibrators and some of the other components are integrated. Tests with five blind and 20 sighted participants of 16 vibrators integrated into a foam insole have found [95]: (i) good recognition of straight-line directions by all but the three teenage blind participants; (ii) poor shape recognition, particularly of diagonal lines; (iii) 100% recognition of five tactile patterns by blind participants, 66% by sighted men and 50% by sighted women. However, the small numbers mean that conclusions about the relative performance of different groups cannot be generalised. A further experiment found that the five best blindfolded sighted and best adult blind participants were able to follow podotactile navigation directions.

The use of a foam insole takes advantage of the good vibrational characteristics of the sole of the foot. Absorption by the foam helps to localise the vibration and prevent it from being transmitted to the whole foot [96]. Incorporating the components into a foam insole also reduces the likelihood of blisters or other irritation. It potentially means that the device can be used with a wide variety of different shoes. However, the insole will need to be an appropriate size to fit the shoe properly and have all the vibrators in appropriate locations, and the best way to achieve this for users with different sized feet seems not to have been discussed in the literature.

### 5.1. Sensors

**Figure 9:**
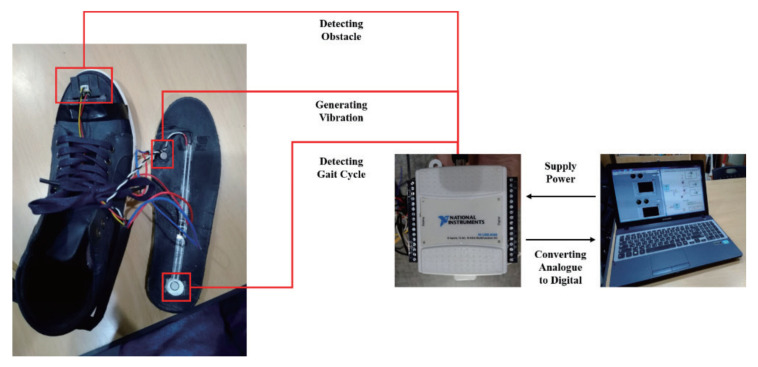
Shoe-worn obstacle and gait detection device [99].

Most of the shoe-based devices provide obstacle detection and avoidance functions, frequently using varying numbers of sonars. Some of them combine this with navigation using tags, such as RFID tags, indoors and GPS and sometimes also a global information system (GIS) outdoors. There are also shoe-based devices that solely provide navigation and devices with additional features. An ultrasonic obstacle detection device with additional functions has four ultrasonic sensors on the shoe to detect obstacles, a water detection sensor to detect wet floors and a 3-axis accelerometer and a 3-axis gyroscope for falls [97]. At least two of the four sonars need to detect an obstacle before the user is alerted. The system has two batteries and switches between them when power drops to 10%. Another ultrasonic device has three ultrasonic sensors on the toecap of each shoe to detect obstacles of different heights and holes in the ground, as well as two mounted centrally above the spectacles’ bridge to detect head-level obstacles [98]. An infrared device uses infrared distance sensors attached to the shoe front and side uppers (see Figure 9) to detect obstacles on the ground [99]. The detection range of 20 to 150 cm is rather short and does not provide additional functionality compared to the long cane. Flexible pressure sensors are attached to the rear of the insoles to provide gait information.

Outdoor navigation devices include a system with GPS on a smartphone and OpenStreetMap GIS to locate the user and calculate the shortest pedestrian route to the chosen destination and the associated waypoints [96]. The prototype uses a cloud server and remote station to facilitate system debugging and development. Moving all the software to the smartphone would reduce privacy and security risks. The combined obstacle avoidance and navigation system has an ultrasonic sensor on the shoe module [100]. GPS, together with Google Maps on a smartphone, is used to determine a path that is constantly updated if the user deviates from it. There is also a sensor for moisture detection. The ability to avoid puddles and spills is clearly useful to blind people. However, this would require either fairly precise information or the user to be navigated around wet patches, similarly to how they are navigated around other obstacles.

A wearable or portable RFID system intended for university campuses [101], but with potentially wider applications, uses high-frequency RFID tags in rooms, halls and outdoor paths. Information about the surrounding area and its precise location is stored on the tag, giving users access to detailed information without the need for external databases. The proposed under-floor tag installation would protect the tags but require additional and potentially more disruptive work than installing onfloor tags. The small RFID reader is integrated both into a cane and the base of a shoe with serial port profile communication to minimise the distance to the RFID tag. This could possibly be replaced by an external antenna on the shoe’s outer edge and electronics attached to the shoe. This would facilitate maintenance and allow use with different shoes. The device also has an ultrasonic sensor on a belt with a range of 3 cm to 6 m to reduce dependence on the long cane for obstacle detection in narrow spaces.

Many of the devices do not include all their components in the shoe. Other locations include spectacles [98], an electronic module attached to the user’s ankle [96], which will be easier to keep unobtrusive for users who wear trousers, a battery-operated microcontroller on a belt pack [98] and an ultrasonic sensor and pager motors on a belt [101].

A slightly different approach involves a thin, flexible metal wire antenna that runs along the shoelace [102]. Its main radiation direction points to the upper front with an angle of about 20°, and it lacks high side lobes and a big back lobe. The system could be used to detect obstacles in front but has not been made into a travel aid through combination with audio or haptic displays to transmit information to users.

### 5.2. User Interface and End-User Testing

**Figure 10:**
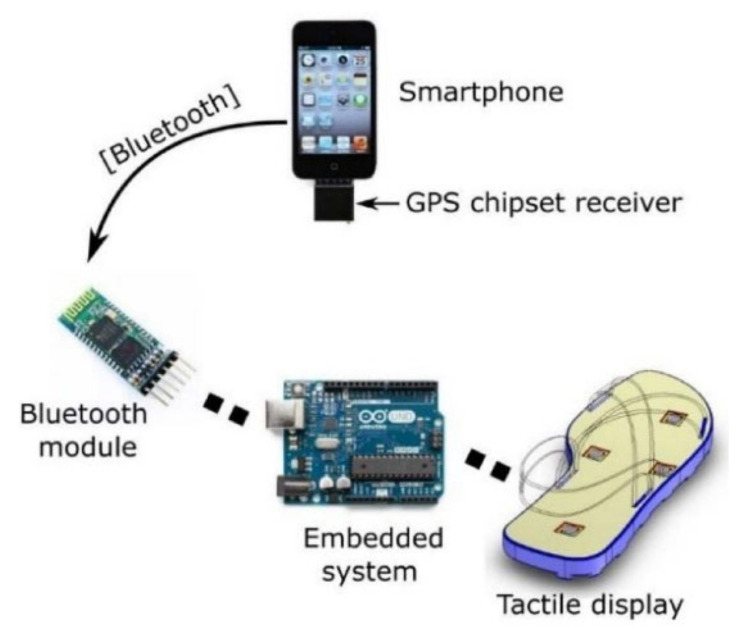
Navigation system with tactile foot feedback [96].

Many shoe-based devices convey information to the user by vibrators on or in the shoe, often embedded in the insole and sometimes in combination with other actuators. This includes four vibrating actuators in a foam insole with the vibration transmitted through dots of epoxy paste that cover the actuators (see Figure 10) [96], three miniature vibrating motors, one for each sensor on the toe caps of each shoe [98], servo motors to adjust the difference between foot and walking direction attached to the shoe front upper [99] and coin vibrators that indicate obstacle distance by vibrational amplitude [97]. An infrared device with an additional gait function uses vibrating motors with intensity varying according to the distance to the obstacle [99]. Tests with 11 visually impaired people found that there was no significant difference in the number of collisions with small obstacles when walking down a corridor using the long cane and the shoe-based device, but significantly more time was required when using the shoe-based device.

An RFID system has 14 pager motors on a belt with the motor chosen to indicate the obstacle distance [101], a much greater number than on other devices. This may cause confusion and could make it difficult to determine which motor is vibrating, so it could be disadvantageous rather than beneficial. There is also an option for vibrational Braille. This may have the advantages of speech in terms of being able to communicate more detailed messages without the disadvantages of blocking environmental sounds. However, the authors recognise the need for extensive testing to see how easy this is to understand while moving. In addition, only a small percentage of blind people are fluent Braille readers. Tests with 20 visually impaired people involved the antenna integrated into a cane rather than a shoe and the use of an audio tone rather than vibrators. Tests against the Locust [103] infrared system found that the RFID system took 30% longer due to the need to find the RFID tags but had no fatal errors in navigation, whereas the Locust system had three.

Some of these devices also have sound or speech output, though this risks blocking environmental sounds. This includes the use of speech on speakers to alert users to obstacle locations and distance, as well as wet floors [97], a buzzer by one of the temples to alert them to head-level obstacles and three miniature vibrating motors in the shoe collar to indicate ground-level obstacles of different heights and holes [98]. The sound of the buzzer could irritate some users.

**Table 5:** Device features and testing for foot-worn devices.

Reference	Where Worn	Functions	Sensors	Feedback	Testing
Abi Zeid Daou et al., 2020 [97]	Foot	Obstacle avoidance, fall and wet floor detection	4 sonars, water detection, 3 axis accelerometer and gyroscope	Coin vibrators, speech over speakers	5 unspecified
Abu-Faraj et al., 2012 [98]	Foot and head	Obstacle detection	3 sonars on each shoe, 2 on glasses	3 mini vibrating motors on shoe, buzzer on temple	1 sighted
Anisha et al., 2021 [104]	Foot	Obstacle avoidance	2 ultrasonic sensors	Buzzer	No end-user testing
Kumar et al., 2021 [100]	Foot and head	Obstacle avoidance and navigation	Ultrasonic sensor on shoes and GPS on phone	Speech output	No end-user testing
Li et al., 2017 [102]	Foot	Obstacle detection	Radiation from shoelace antenna	Not yet added	No end-user testing
Velazquez et al., 2018 [96]	Foot and ankle	Navigation	GPS on smartphone	4 vibrating actuators	20 sighted tactile pattern recognition 2 blind outdoors with long cane
Manikandan and Hussain, 2017 [105]	Foot	Obstacle avoidance	Ultrasonic sensor	Vibrating motor	No end-user testing
Willis and Helal, 2005 [101]	Foot and waist	Navigation and information on university campus	RFID reader in base of shoe, sonar on belt	14 pager motors on belt and vibrational Braille	20 visually impaired users
Yang et al., 2018 [99]	Foot	Obstacle avoidance	Infrared sensors	Vibrating motors	11 visually impaired, compared to long cane

## 6. Responses to the Three Research Questions

### 6.1. The Important Design Issues in Wearable Travel Aids and the Extent to Which They Are Taken Account of in Different Devices

Many of the design factors considered in the context of wearable medical devices [106] are also relevant to wearable travel aids and overlap with those suggested by [41,42,55]. Drawing on these sources, adding some user-related factors and editing conditions related specifically to obstacle avoidance, wireless etc. gives the following:

- Form: small size, lightweight, unobtrusive and attractive.
- Use: easy to understand and use user interface, real-time response, sufficient/appropriate environmental information, long battery life and easy recharging.
- Wearability and reliability: stably attached to the body, not affecting body movement, comfortable to wear, safe in contact with the body, robust to different climatic conditions, reliable.
- User factors and context: age, gender, language/culture, available infrastructure, low cost, value for money.

The devices considered here are all prototypes. There are, therefore, issues of what needs to be designed in from the start and what features can be modified and improved with further iterations over time. A summary of device properties is presented in Table 6 at the end of Section 6.1.4.

#### 6.1.1. Form

An appropriate device appearance is generally vital for user acceptance. In addition, blind, just as sighted people, use appearance to present themselves in a particular way. However, appearance has received limited attention in most of the devices. Devices such as Uasisi [73] and Vista wearable [74], which can be attached to pockets or integrated into other wearables, are probably the most successful at being unobtrusive.

Some of the devices worn on belts, wrist bands and gloves have the potential to be unobtrusive, but this will require some of their components to be made smaller. Vest-worn devices can be unobtrusive if they can be worn under clothes and the materials are not bulky. If required to be worn on top, then issues of appearance and style become important, and there would be advantages in designing the components in one or more small enclosures that can be attached to clothing. Shoe-worn devices with components included in foam insoles are unobtrusive. However, such insoles are likely to be unsuitable for use in very narrow shoes or shoes with high heels.

**Figure 11:**
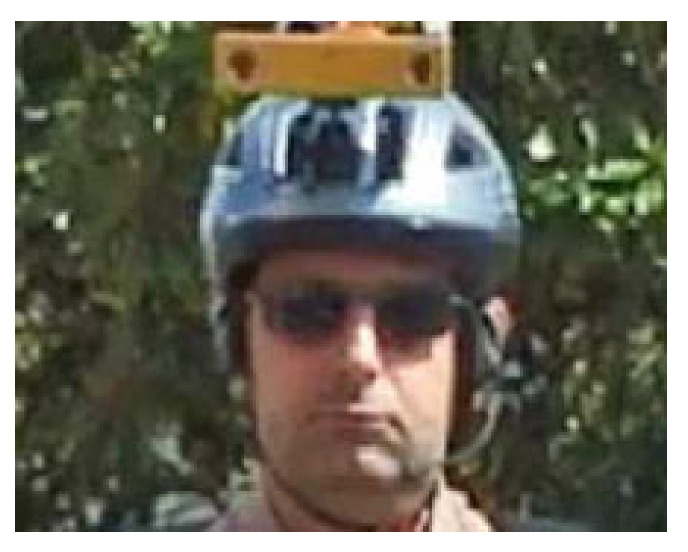
Navig helmet.

Their position and visibility make the appearance of head-worn devices particularly important, but this seems to have received minimal attention. There is both a need for all components to be miniaturised and the headgear or spectacles they are worn on to be reasonably attractive and in line with the user’s desired image. This makes, for instance, helmet-worn devices, such as Navig (see Figure 11) [57], unsuitable for many potential users who would probably prefer not to be seen in public wearing a helmet. While many blind people wear dark glasses, these are frequently chosen for their appearance, and users may not be willing to replace them with a travel aid on another pair of glasses. Thus, there is a need for device components that can be attached to a diversity of headgear and spectacles and may mean some components being attached to, for instance, belts rather than head mounted.

A few devices, such as [54,89], include laptops in backpacks. This additional weight can, in many cases, be reduced by transferring software to smartphones, other mobile devices or chips and some authors, e.g., [49], suggest this. A few authors mention device weight, and others mention devices being small and lightweight, without specifying a particular weight or discussing making their devices small and lightweight. However, there is no discussion of what this means in practice and what weight can be comfortably worn on different parts of the body over an extended period.

#### 6.1.2. Use

Most devices provide audio or tactile output to the user or a combination of the two, but there are a few exceptions where an output display has not yet been added and plans to do so are not discussed [58,102]. In the case of Navig [57] and a smartphone obstacle detection and classification system [42], the authors indicate that this is being or will be developed and involve audio without blocking environmental sounds. The other devices involve a variety of different audio and tactile displays or sometimes both. Tactile displays will be discussed first.

Of the devices presented, only the intelligent glasses [45] provide an active display. This has the advantage of allowing users to explore freely, in this case, a tactile map of obstacle locations. However, there is likely to be a learning curve and the possibility that some users may experience difficulties in learning to use it. Other questions relate to whether the display can be used while walking or if the user will need to stop to do this, and how easy it is to learn to explore the display in a way that ensures the closest obstacles are explored first and that none of them are missed.

The other tactile displays are all passive and have varying numbers of vibrating motors. The simpler displays use one to four vibrators located on different parts of the body. An overhead and ground-level obstacles detection system has three vibrators on the fingers to indicate near, middle and far distance obstacles. Other simple displays, frequently involving four vibrators, provide the travel direction and instruction to stop and scan, e.g., [54,80]. An RFID indoor navigation system has 14 vibrating motors on a belt to indicate distance [101], which seems over complicated. End-user tests involved an audio alert rather than the vibrating motors. A glove-worn device also has 14 (tiny) vibrating motors [90] but does not state how they are used or why so many are required. One output display that users may find complicated involves seven solenoids on a wristband, with the left and right ones indicating the side and the others the obstacle angle in a binary number system [89].

Many of the speech and non-speech audio displays have simple, relatively easy to understand output. Using one audio parameter to convey information, for instance, faster beeping to indicate a closer obstacle [59], is generally comprehensible, though users may need a bit of experience to determine the beeping speed at which they need to take avoidance action. Sonification schemes, e.g., [46,52], may be more difficult to learn. Experience with vOICe [41] shows promising results after extensive training, but not all users may be able to engage in extensive training and concentration on sonification schemes could divert attention from important environmental sounds.

Only a few devices provide user input, probably due to the nature of their functions and the lack of options for the user to control. This is generally relatively simple to use, involving tapping a screen once or twice, e.g., [53], or speech, possibly with Dragon Naturally Speaking speech recognition [57]. However, [88] requires arm movements, which users with poor coordination or other impairments may find difficult, and in [49], the user is required to stop to touch the phone touchscreen to receive object information.

Devices need to respond in real-time to be useful, i.e., to provide output sufficiently fast to users so that they can use the information to avoid obstacles or make other travel decisions. The ultrasonic sensors used in the devices discussed have a maximum range of 6 m and, consequently, less than 0.036 s obstacle response time. Analogous arguments can be made for infrared systems. Since most ultrasonic devices do not require very heavy signal processing, their developers have generally not considered it necessary to mention or discuss a real-time response. Camera vision devices generally require considerable processing, giving rise to the issue of whether they can carry out real-time processing. A common walking speed is 1.42 m/s. Blind people frequently walk more slowly than sighted people, but some walk faster but are unlikely to do so at greater than 2.1 m/s. This implies a response rate of 1.42 fps should be sufficient and 2.1 frames per second (fps) definitely so, but 7–10 fps is preferable. A number of authors provide response times, mainly in frames per second but sometimes in other units. Most of them have reasonably good response rates, but Sveta [46] has one of less than 1 fps, and an RFID device [101] has rates that reduce as the amount of data in the tag increases and which may be as low as 1 tag per second. There may be some trade-offs between acceptable reductions in speed and increases in safety. However, devices with a too slow response rate will either slow users down, possibly leading to abandonment, or compromise their safety.

What is sufficient or appropriate environmental information generally depends on the context and what the user wants the information for. There are also trade-offs between the amount of information that can be provided and the need to avoid cognitive overload. There is also the risk that focusing on information from a device could distract the user’s attention from important (audio) environmental information. It is also recognised that blind people generally use all their senses to obtain information, though (like sighted people), some blind people are better at this than others. Therefore, there is value in device design to provide information that is complementary to that available from the other senses, but this seems not to have been considered.

Having to change device batteries while out and about is clearly highly undesirable. A duration of eight to ten hours between charges would allow users to spend time visiting and travelling around an area. A duration of one to two hours would cover a long commute. Watch your Head [75], the virtual white cane network [71], and a necklace sonar [72] meet the all-day requirements and a few other systems, e.g., [62,77], would allow a good half-day use. However, in some cases, e.g., [90,97], further work is required to extend battery life. A few authors mention low power but do not provide details. It is important that the device is rechargeable on the grounds of both the environment and cost. An ultrasonic body-worn device uses a rechargeable battery [72], and the development of electromagnetic recharging while walking has been proposed for a shoe-worn device [105]. This would be an interesting option, but unfortunately, no details are provided.

#### 6.1.3. Wearability and Reliability

The devices generally seem to be stably attached to the body on belts, bracelets, bands, gloves, vests, insoles, spectacles and headwear. However, there has been no discussion of ensuring that, for instance, bands, belts, bracelets and glasses do not break or become too loose and fall off. However, devices such as Vista wearable [74], which can be clipped to armbands, clothing and pockets, need to be particularly securely fastened to them, as otherwise, they could easily come off and possibly also be lost.

The discussion of device design has generally not considered wearing comfort. This is affected by factors such as the materials used and appropriate sizes, e.g., vests, bracelets, belts, bands and glasses so they fit comfortably without moving around. Only in the case of the ActiveBelt [70] has an adjustable design been considered, in this case, through the use of elastic rubber parts. The authors, unfortunately, do not comment on how well this works in practice and what range of waist sizes can be accommodated. The authors of [89] have based the size of the wrist band on the size of one of the authors’ wrists and the solenoids used. However, they do not discuss how the band could be adapted for users with other wrist sizes. In the case of other devices, there has been no discussion of the advantages and disadvantages of adjustable sizing and several size options. Sizing also affects the appearance and the position of sensors and tactile displays. For instance, fixed-position sensors and vibrating motors intended to be at the sides of belts or wristbands could be at the front or back, bands that are too large may not stay in place, and those that are too small may be uncomfortable and constraining or impossible to put on. These factors may make it more difficult to investigate adjustable or multiple size options at a later stage. A tactile glove uses mechanical vibrators [90] to avoid the possibility of pain from electrotactile vibrators [107]. However, this issue has not been considered more widely in the devices surveyed.

There has been limited consideration of the possible impacts of the device on body movement, and only a few authors report user comments on this. For instance, [73] found that (blindfolded) participants preferred the device to be attached to an ankle rather than a wrist band, as this gave a more natural way of moving and did not require users to move their arm around to try to sense obstacles. The authors of [86] found that their bracelet-worn device ‘put significant constraints’ on participants’ hand and body movements in a cluttered environment. On the other hand, Ref. [74] found that participants preferred a similar device to be wrist-worn to support active scanning.

Many devices use off-the-shelf components to reduce costs and where different options are available, as is frequently the case, they are likely to prefer components of proven reliability. Authors generally do not provide information on the standards met by the components used or the measures taken, if any, to improve reliability. However, [97] use redundancy to improve reliability, including obstacle detection by two out of four sonars before the user is alerted and the provision of two batteries with switching between them. It is important that users do not receive over-frequent and unnecessary alerts, which could distract their attention and mean they miss some obstacles, and that they are alerted to obstacles that they could collide with. Extensive testing would be required to determine whether the need for confirmation could lead to users not being alerted to some obstacles. Ref. [97] also carry out diagnostic tests and provide an alert that the system is no longer functioning if problems are encountered. However, there is no discussion of wider safety issues of devices in contact with the body or clothing (e.g., Ref. [106] for more information). In addition, reliability is the ability to perform well and consistently over a (significant) period of time with all users, but user testing has generally been for (very) short periods and only involved relatively small numbers of users.

The discussion of device development does not seem to have considered the need to operate in a wide range of climatic conditions. However, the impact of different lighting conditions has been considered for a few of the camera vision systems. It is not relevant to the ultrasonic and infrared systems that produce their own radiation beams. The Sound of Vision [51] uses slightly different processing approaches in different lighting conditions and has reduced functionality in poor lighting conditions, both indoors and outdoors. The authors of [58] found that where buildings block the sunlight, the images were over or underexposed, but this seems not to have affected performance to any significant extent. The authors of [49] obtained slightly reduced accuracy outdoors compared to indoors and reduced accuracy in both cases for night scenarios, with the lowest accuracy of 97.9%.

Since wearable devices are close to or make direct contact with the user’s skin, it is important to avoid materials that may cause irritation or be allergenic to some users, such as rubber latex. The materials used will also affect user comfort, particularly when in direct contact with the skin. Although not directly related to safety in contact with the body, some users may have ethical objections to particular types of materials, such as leather, and are unlikely to use devices that include them. However, the only authors who mention the materials used are [70], and they unfortunately, use both rubber and leather.

#### 6.1.4. User Factors and Context

Blind people are found throughout the world, with a much higher percentage of the population being blind or partially sighted in low- and middle-income countries [1]. However, there seems to be a tacit assumption that devices will be used only in countries that have advanced modern infrastructure. This reduces device usefulness on a global scale. A number of devices provide speech output, but only [60] specify the language(s) used, in this case, Bengali and English.

The authors of [77] mention the different attitudes of older and younger blind people to trying the device: younger people were interested in using the system, and older ones, at least initially, preferred to rely on their own senses. However, issues relating to different groups of users possibly having (slightly) different requirements and device design to meet the needs of the full diversity of potential users have been given minimal attention in the literature.

There is limited information about ease of use since end-user testing, when carried out, has generally focused on performance and rarely had a qualitative element, asking about factors such as comfort and ease of use. However, there are a few exceptions. For instance, an indoor and outdoor object detection and navigation system [53] was considered useful by 10 blind participants and easy to wear by nine of them. Only a few authors provide information about device costs, and a few others indicate that their devices are low cost without mentioning a specific amount. However, it should be noted that there could be a significant difference between the costs of a prototype and a marketable device and what is affordable varies significantly, particularly between the Global North and South. The needs for blind people to have higher incomes and more state funding for assistive devices are beyond the scope of this work.

**Table 6:** Device properties.

Reference	Battery Life, Power	Low Weight	Small Size	Real Time	Low Cost
Abi Zeid Daou et al., 2020 [97]	40 min	x	x		x
Abu-Faraj et al., 2012 [98]		x	x		x
Agarwal et al., 2017 [59]		x	x		x
Alayon et al., 2020 [89]		x	x		
Anisha et al., 2021 [104]		x	x		
Bai et al., 2019 [53]		x	x	x	
Balakrishnan et al., 2007 [46]		x	x	0.8 fps	
Bharathi et al., 2012 [62]	3–4 h	x	x		
Bhatlawande et al., 2013 [86]		x	x		
Brilhault et al., 2011; Katz et al., 2012 [56,57]		x		15 fps	
Brock et al., 2014 [92]		x	x		
Caraiman et al., 2017 [51]		x	x	10 fps	x
Dakopoulos, 2009 [55]	low power	x	x	15 fps	
Diaz et al., 2020 [80]	2.5 h	x		x	
Everding et al., 2016 [52]		x	x	20 fps	
Fiannaca et al., 2014 [50]		x	x		
Fusiello et al., 2002 [44]		x	x		x
Gao et al., 2015 [71]	9.5 h		x		
Garcia-Macias et al., 2019 [73]		x	x		
Gay et al., 2020 [81]		x			
Hsieh et al., 2020 [82]		x			x
Huang et al., 2017 [93]		x	x		
Jameson and Manduchi 2010 [75]	90+ h	x	x	x	
Khampachua et al., 2016 [88]		x	x		
Kuc, 2002 [87]		x	M	x	
Kumar et al., 2021 [100]		x	x		
Laubhan et al., 2016 [61]	low power	x	x		
Lee and Medioni, 2014 [54]				28.4 Hz	
Leung et al., 2014 [58]		x	x	30 fps	
Li et al., 2016 [79]			x		
Li et al., 2017 [102]		x	x		
Lin et al., 2019 [49]		x	x	x	
Linn et al., 2017 [85]		x	x		
Mancini et al., 2018 [91]		x	x		
Manikandan and Hussain, 2017 [105]		x	x		x
Mattoccia and Macri, 2014 [48]		x	x	20 fps	
Meijer, 1992; Dakopoulos and Bourbakis, 2009 [41,47]					x
Mocanu et al., 2016 [77]		M		10 fps	
Molina et al., 2015 [74]		x	x		
Pradeep et al., 2010 [63]		x		x	
Prathipa et al., 2019 [69]		x	x		
Riehle et al., 2013 [83]		x	x	x	
Sayed et al., 2020 [65]		x	x	x	
Shoval et al., 1998 [68]				x	x
Tanveer et al., 2015 [60]		x	x		
Tapu et al., 2013 [78]		x		7 fps	
Tsukada and Yasumura 2004 [70]		x	x		
Velazquez et al., 2006 [45]		x	x		
Velazquez et al., 2018 [96]		x	x		
Venkateswar and Mehendale, 2012 [67]		x	x		
Villamizar et al., 2013 [72]	8 h	x	x		
Willis and Helal, 2005 [101]		x	x	>1 tag/s	
Yang et al., 2018 [99]		x	x		
Yeboah et al., 2018 [76]	0.4 W	x		x	
Zelek et al., 2003 [90]	1 h	x	x		x

### 6.2. The Relationship, If Any, between Where and How Travel Aids Are Worn and Their Design, Features and Functions

Most of the wearable devices that use cameras are head or chest-mounted. This avoids many of the difficulties that would otherwise occur with focusing the camera and ensures that it is automatically aimed forward. Cameras on head-mounted devices, particularly spectacles, have approximately the same field of view as a sighted person. It is relatively easy to scan the environment through head movement, whereas chest or waist-mounted cameras require body rotation for scanning [63]. Ultrasonic sensors are used in devices worn on all parts of the body, including the feet, and mini devices that can be integrated into or pinned to clothing. Incorporating sonars in a wearable device potentially allows the use of a larger number of them, giving a much greater angular coverage, particularly if they are located on a belt or the chest, e.g., [67,68,77].

The majority of glove and bracelet-worn devices use sonars. Where they use cameras, they are chest or waist mounted, e.g., [91], rather than attached to the glove. Shoe-worn devices mainly use sonars, sometimes with GPS, e.g., [96], or other sensors, e.g., [100], and do not use cameras. This indicates particular design issues with camera vision and a relationship between head and chest-worn devices and camera use. Some of the devices that use cameras on a smartphone attach the phone to the body, e.g., [42]. Where sonars are used on head-mounted devices, they are sometimes also attached to spectacles, for instance, [59]. Mounting travel aids on glasses seems to be the most commonly used solution for head-mounted devices. Since many blind people already wear (dark) glasses and other forms of headwear can occlude the ears and interfere with the perception of environmental sounds, this has advantages.

Head-mounted devices most commonly use sound displays, possibly due to the lack of obvious sites on the head for tactile stimulation. There is also the risk that repeated vibration could cause at least some blind people headaches or distract their attention from environmental sounds. Where tactile displays are used, sometimes together with audio displays, they are worn on other parts of the body, such as on a vest [54], armbands and the back of the neck [48]. The exception is tongue stimulating displays, e.g., [64], though they have not yet been used in any travel aids. The tongue is very sensitive, but devices worn in the mouth may have poor user acceptance due to, for instance, negative impacts on the appearance, possible impacts on speech and the need for very careful cleaning.

Body, hand and arm and foot-worn devices generally have tactile feedback displays. This is particularly the case for devices worn on vests, belts, arm-bands or bracelets, gloves and foam insoles. This may be due to the reasonable surface areas of the body available for such devices to stimulate. However, sensitivity varies and is much greater on the hands and soles of the feet than on the torso and wrists [94].

Most wearable devices, regardless of where they are worn, provide obstacle detection and avoidance functions. Some also provide object recognition, navigation to a destination or descriptions of the surrounding area. Other than the Navbelt [68], which uses eight sonars, all the environmental representation and description systems seem to involve head-mounted cameras. This is probably not surprising due to the advantages of camera vision systems in potentially being able to obtain full scene overview information. Head mounting has advantages in giving a similar visual field to that of a sighted person and only requiring the user to turn their head in order to scan the scene [69]. However, use of this information depends on the effectiveness of the processing algorithms used to analyse it and, for instance, extract and identify objects, and the audio or tactile display used to present it to the user in a comprehensible format without cognitive overload.

Some shoe-worn devices provide additional functions, such as the detection of moisture, wet floors or falls and gait monitoring [97,99,100]. Using shoe-based sensors for detecting wet floors has the advantage of proximity. Using shoe-based sensors for gait and fall detection is also logical, though fall detection sensors could be used on other parts of the body. A shoe-worn RFID campus information system [101] has the advantage of the nearness of the RFID reader to the (under floor) tag. Some hand and arm-worn devices provide other functions, such as route learning from a tactile map using a tactile wristband [92] and a traffic light indicator on a glove [93]. Since the tactile map device uses a finger to explore the map, the use of a hand or arm-worn device, such as a wristband device, is logical. However, there seems to be no particular reason for the traffic light indicator to be on a glove.

### 6.3. Limitations of Existing Devices, Gaps in Provision and Future Research Directions, Particularly with Regard to Meeting Potential Users’ Needs

Most wearable travel aids have obstacle detection and avoidance functions. Some of them, e.g., [50], provide information on high/overhanging obstacles and others, e.g., [86,88], have a much longer range than the long cane; therefore, they are potentially able to provide preview information and alert users to obstacles at a distance. However, many devices seem to provide only standard obstacle avoidance functions with no additional features compared to the long cane. In addition, there has been minimal discussion, analysis or testing of how the features and performance of these devices compare with those of the long cane. In a few cases, comparative testing of the device and long cane performance has been carried out, e.g., [49,99], but the relatively small number of participants and limited routes used mean that the outcomes could change if further testing were carried out. There are also issues of user acceptance of devices with similar functionality to the long cane, but which are more expensive and complex and also lack the long cane’s user protection function of automatically keeping users at arm plus cane length from any detected obstacles.

Wearable devices have an analogous spread of functions to non-wearable travel aids, with the advantages of keeping the hands free, in some cases being more discrete, and not necessarily needing to be searched for after a pause in use, as some of them can be left on the body or put in a pocket when not in use. In addition, devices with spectacles or other head-mounted cameras reduce the difficulties in focusing the camera, can scan the environment through head movements and are better suited to providing high-quality environmental information than purely portable devices. However, to date, wearable devices have not been developed to try to fill the gap in the provision of support for the large section of the blind and partially sighted community who only go out accompanied.

Device design and development have generally not considered privacy and security issues. However, blind people could be targeted as a result of being perceived as particularly vulnerable. Devices that transmit location or other information to a server, another device or a third party have a risk of interception. Bluetooth links used, for instance, in [69,74,88], are considered vulnerable [108]. Devices with wireless capability could be attacked, as has happened with pacemakers [109]. Analogous attacks could affect the functioning and reliability of travel aids for blind people, with negative impacts on user safety. There is also the risk of unauthorised access to data if a device is compromised, even if it is not shared [110]. Particular risks relate to the generation and store of location data and route information and the camera-generated images of the surroundings, which could possibly be used to identify the user’s location. Wrist-worn travel aids that include accelerometers could capture hand movements when entering keypad information [107]. This is an infrequently used design, with exceptions including [88], and there are probably advantages in it not being adopted to a large extent. There may be a risk of cameras capturing images of keypad entry, but this would require the camera angle to be deliberately changed from straight ahead to down. Generally, poor ATM accessibility has reduced use by blind people with the unexpected benefit of reducing this risk.

Camera vision devices also pose a risk to the privacy of bystanders, as their images could be captured. The fact that blind device users are frequently unable to aim the camera to exclude such images may increase this risk. Images may also be captured by camera-based devices used to identify the presence, location and number of other people as part of obstacle avoidance. There is some evidence that bystanders have greater acceptance of head-mounted devices used for assistive purposes, though this is counter to users’ desire for others not to know they are using an assistive device [111]. Since the focus of this paper is travel aids, the privacy issues related to the capture of other people’s images to support social interaction are beyond its remit.

A number of devices provide audio feedback to users. However, only a few of them, e.g., [48,57,77], use bone conduction earpieces or headphones to prevent the ears from being covered and masking environmental sounds. Consequently, users of these devices will have considerably reduced access to the environmental sounds that they require for safe and effective mobility, and this could negate the value of the device. In principle, it should be possible to replace existing earpieces and headphones with bone conduction versions, though this could affect the cost, and some design modifications may be required.

Tactile devices and those with non-speech sounds can be used by blind people across a wide range of countries and cultures. However, there seems to be a lack of research on any differences in interpretation of sounds and vibration across countries and cultures and whether this could affect the use of travel aids. Speech output needs to be in a language the user understands and preferably the one they think in. This is particularly important for warnings/urgent alerts, where even brief delays in processing information or misunderstandings could have serious consequences. However, there seems to be no discussion in the literature on wearable travel aids of making devices available in multiple languages, and only [60] mentions the language of speech, in this case, Bengali and English.

Few authors have actively involved blind people in device design and development, with the exceptions including [57]. The involvement of potential end-users in device design and development is essential, as they are the only people who understand their needs and preferences. Otherwise, device design can easily become technology driven, and the results not meet the needs or otherwise be unsuitable for potential blind end-users. There is also a need to involve users with diverse characteristics with regard to age, gender, ethnicity, history of vision impairment and other factors. The authors of [57] involved both early and late blind people, people of both binary genders, though women were underrepresented, and a range of ages, though people over 60 were not represented, and they do not mention ethnic diversity.

Device testing, at least as reported in the literature, has been limited. There has generally been a lack of combined laboratory/functional and extensive end-user testing. Testing with blind and partially sighted people is essential to determine how devices perform in real situations and what blind people think of them. However, end-user testing has only been carried out for some devices and has, in a number of cases, only involved blindfolded sighted people, e.g., [54,71,92]. Where testing has involved both blind and sighted people, differences in performance have sometimes been obtained, e.g., [93]. In addition, sighted people generally rely very heavily on vision and may experience difficulties in adapting to walking blindfolded. Therefore, the results of tests with blindfolded sighted people cannot necessarily be generalised to blind people. The number of participants has frequently been small, with testing involving only one to six people in several cases, e.g., [61,85,97].

All the devices discussed are prototypes, and the literature seems to lack evidence of moves to further development and commercialisation or non-commercial means of distribution to users. As already discussed, the devices are in various stages of development; many of them require considerable further work, and some may not offer sufficient benefits compared to the long cane or otherwise do not meet user needs. However, prototypes that are not fully developed and commercialised or otherwise made available to users are of no great use to them.

#### 6.3.1. Suggested Device Improvements

The discussion of device design issues and limitations in Section 6.1 and Section 6.3 will be used to provide a framework for suggesting improvements to devices. Design issues have been divided into the categories of form, use, wearability and reliability, and user factors and context. The main aspects of form that could be improved are appearance, size and weight. Appearance is important for all devices, but particularly so for head-mounted ones. Design to allow devices to be attached to spectacles or headgear of the user’s choice, as in [51], would give the user control over their appearance when wearing the device. The appearance of several devices could be improved by the device or particular components being made smaller and in some cases, also lighter. This would make the device less obtrusive.

Use areas where improvements are possible include the number and position of vibrators, battery life (between charges) and speed of response. The systems in [90,101] both have 14 vibrators. It seems unlikely that most users will be able to distinguish the different vibrator positions and use all the vibrators effectively. Therefore, reducing the number of vibrators could give an improvement. However, end-user tests with different numbers and positions of vibrators will be required to determine the best option. Several devices have batteries with a relatively limited duration between charges. A duration of 8–10 h would be desirable to enable all-day use and a minimum of 2 h for, for instance, a long commute. A combination of design changes to reduce power consumption, the addition of a power management system and changing the type of battery could be used to improve battery duration. However, any changes to the battery should not increase its size, weight or cost (other than minimally). Where camera vision devices do not provide a sufficiently fast/real-time response, e.g., [46], performance could be improved by modifying the design to give a faster response time.

Wearability could be improved by making some devices smaller and lighter and ensuring that the garments or other wearables they are attached to fit well. Devices such as [54,89] could be improved by using software on a mobile phone or other mobile devices rather than a laptop in a backpack. This would reduce device weight and size and avoid the need to carry a backpack unless required for other items. Appropriate sizing is important for wearing comfort and appearance, ensuring that sensors and actuators are appropriately positioned and that belts and wristbands stay in place and do not fall off. However, most of the devices provide only one size version that cannot be adjusted. Thus, providing different sizes or options to adjust the size, as in [70], would improve wearability. This will need to be done in a way that ensures that all sensors and actuators are in appropriate positions so that the user obtains correct information. The feasibility of providing different size options will depend on the number of users, which to date has been low for electronic travel aids. As already indicated, there would be advantages in designing head-worn devices to be attached to headgear or spectacles chosen by the user. A similar approach could potentially be used with devices worn on other parts of the body. This is already the case for [73,74], but they are relatively simple devices. More complex devices with multiple sensors and actuators would require users or an assistant to attach them in appropriate positions to ensure correct information is conveyed to users. Several shoe-worn devices have components integrated into an insole. While potentially allowing use with a variety of shoes, there are still issues of the appropriate insole size and whether it is feasible to offer different size options.

Reliability is clearly critically important, as (unexpected) loss of function or errors could significantly reduce user safety. Devices should also be able to operate in a wide range of climatic conditions. However, minimal information is available about the measure to ensure reliability and good performance in different conditions. Therefore, in many cases, improvements could be obtained by the use of design redundancy and backup/failsafe mechanisms, but without adding more than minimally to size, weight and cost. Device casings should be waterproof and components designed to operate over a wide temperature range. Camera vision devices should ensure good performance in different lighting conditions, if necessary, by using slightly different processing approaches, as in [51]. Device materials should be non-allergenic and vegan friendly. For instance, the leather and latex in the belt in [70] should be replaced by non-allergenic vegan-friendly materials.

Wearable travel aids are potentially of interest in many different countries and cultures. However, the general lack of indication of which language options are available probably implies that speech output devices only have one language, generally English. Therefore, options for language choice for devices with speech output would be an improvement. Another possible improvement would be the inclusion of customisation options to enable devices to be better tailored to particular groups of end-users. This should involve participatory design and end-user testing with different groups of end-users. The same is true of investigating improvements to ease of use, where optimising the number of vibrators has already been mentioned. Extensive end-user testing with large numbers of users with diverse characteristics is required for all the devices, though a reasonable amount of testing has already been carried out for some devices. There is also a need to move to final versions that can be commercialised. The use of participatory design approaches that actively involve potential end-users is likely to lead to considerably improved outcomes.

The limitations discussed in Section 6.3 include limited functionality with only basic obstacle avoidance functions with no additional functions; the lack of devices for blind and partially sighted people who only go out accompanied; the lack of privacy and security features; and the use of standard headphones or earpieces that block environmental sounds. Devices with only basic obstacle avoidance functions could be improved by adding functions for detecting high/overhanging obstacles or obstacles at a distance. Comparative testing against the long cane should also be carried out using both qualitative and quantitative data.

Filling the gap in the provision of devices for blind and partially sighted people who only go out accompanied will probably need the design of new devices, preferably using participatory design approaches, not the modification of existing devices. Privacy and security are potentially relevant to all electronic devices but particularly important for those that transfer data to another device or generate and store personal data, such as location and route data. Potential improvements include adding a privacy management system and design modifications to reduce the risk of data interception. This should be followed by intensive testing against simulated attacks. The addition of a face recognition and exclusion feature could be used to prevent camera vision devices from capturing bystanders’ faces. This will reduce potential privacy violations that could be experienced by bystanders but could make the system more complicated. Replacing standard earpieces of headphones by bone conduction ones can enable access to environmental sounds.

This gives the following types of improvements:

*Form and wearability*

- Making devices smaller and lighter, including replacing laptops in backpacks with software on a mobile device.
- Designs that allow the device to be attached to spectacles, shoes, headgear or other clothing of the user’s choice.
- Different size options or adjustable sizes.

*Use and functionality*

- Additional functionality, e.g., detection of obstacles at a height and distance for devices that only provide basic long cane type functionality.
- Increasing speed/making device real-time.
- Improved battery life through, e.g., power management or improved design.
- The use of bone conduction headphones for devices with audio output.
- Multiple language options for speech output systems.
- Optimisation of the number of vibrators.
- Other customisation options.

*Privacy, security and reliability*

- Improved privacy and security management, including privacy management systems and design to reduce the risk of data interception.
- Face recognition and exclusion function for camera vision devices to prevent privacy violations for bystanders.
- Backup/failsafe options to improve reliability.
- Adaptation to different climatic conditions, including waterproofing and components that can be used in a range of temperatures.
- Adaptation to different light conditions for camera vision devices.

The improvements in the first two categories that could benefit different devices are presented in Table 7 and Table 8. The symbol ‘?’ is used in Table 7 to indicate a lack of information in the reference(s) on whether or not the device already includes this potential improvement. The potential improvements in the third category (privacy, security and reliability) have not been tabulated, as very little information about these areas is available in the references. It is, therefore, possible that some of these issues have been into taken account in some of the devices but not discussed in the literature. The provision of customisation options has not been included in the table as most devices could benefit from them, but this has also rarely been considered. There may be trade-offs against costs and other factors.

The differences between suggestions for improvements and device evaluation should also be noted. In particular, there is no obvious relationship between device quality or performance and the number of suggested improvements and devices with fewer suggested improvements should not be assumed to be better than those with a larger number. There may be trade-offs between some of the suggested improvements and other factors, such as cost and size, or other factors that make some of the suggested improvements inappropriate for some of the devices. Finally, it should be noted that these are purely suggestions. It is not claimed that these suggestions will lead to improvements in all cases, though overall, this is likely.

There are a number of other potential improvements that are presented for specific devices but may be more generally relevant. Some devices, e.g., [58], do not mention feedback to users; [78] plans to add it, and [102] does not yet include it. Adding appropriate feedback that uses bone conduction headphones for audio, provides a choice of languages for speech, and an optimised number of vibrators for vibration would be a useful improvement. The authors in [93] do not indicate how information is obtained from traffic lights, though there are presumably sensors or some other mechanism for doing this. Another issue [58] is the use of poor-quality image sensors. This could be improved by the use of better-quality sensors while taking into account the need to keep costs low. It is possible that several other devices could benefit from the use of better-quality sensors, while recognising the need to keep costs low.

#### 6.3.2. Suggestions for Future Research Directions

The answers to the three research questions indicate that considerable further research is required and suggest the following research directions, which have been organised into two groups.

*End-user-led design and development (and commercialisation) of wearable travel aids*:

- To support at least some groups of blind and partially sighted people who currently do not travel unaccompanied in taking at least short trips in their local area on their own.
- To support indoor navigation, particularly travel around large complex buildings and groups of buildings, such as hospitals and university campuses.
- To support more precise outdoor navigation, including building entrances, possibly based on high precision GPS systems.
- To support the last 10 or 20 m of travel to a building entrance. The development of more precise outdoor navigation systems may remove the need for this.
- With multi-lingual speech output where speech is used.

*Other research:*

- Extensive testing with blind people, including comparative testing against the long cane and the combination of the device and long cane against either on its own.
- Investigation of the advantages and disadvantages and relative performance of the different approaches to processing the output of camera sensors for use in wearable travel aids.
- Investigation of the advantages and disadvantages and relative performance of the different approaches to improving GPS precision for use in wearable travel aids.
- Privacy and security issues in wearable travel aid design, both at the individual device level and as more general solutions.
- The impact, if any, of gender, age, ethnicity and other demographic factors and how they should be taken into account in device design.
- Cultural and other issues related to the perception of vibrotactile and other tactile stimulation and non-speech sounds and any possible effects on the use of travel aids.
- The trade-offs users are willing to make between device performance/functionality and other factors, such as reduction in walking speed and appearance.
- Whether there are any advantages in combining sonars and camera vision sensors.

## 7. Conclusions

The paper has provided an overview of the different types of wearable travel aids used to support blind people with a focus on issues related to design and wearability. Its main contribution is the response to the following three research questions:

1. The important design issues in wearable travel aids and the extent to which they are taken into account in different devices.
2. The relationship, if any, between where and how travel aids are worn and their design, features and functions.
3. Limitations of existing devices, gaps in provision and future research directions, particularly with regard to meeting potential users’ needs.

The responses show that, with a few, often partial exceptions, the focus has generally been on developing travel aids rather than specifically considering the wearability requirements of wearable travel aids. This means that device development has not fully taken into account the potential of wearable devices. It also means that issues related specifically to wearability, such as the need for devices to fit people of a variety of different sizes and for long-life rechargeable batteries, have only occasionally been considered. Size affects comfort, appearance and the position of sensors and tactile displays, so they cannot necessarily be corrected easily at a later stage. This is just one example of the differences between designing wearable and portable travel aids.

There seems to be a move to increasing use of camera vision rather than other types of sensors. However, there is also a suggestion that combining camera and ultrasonic sensors can improve performance, which is worth further investigation. The move to camera sensors has led to a prevalence of head-mounted devices as the best position in terms of focusing and scanning the environment. However, there are a number of other interesting devices, including several worn in shoes.

The survey has shown both that a lot of very interesting work has taken place and that it, unfortunately, has a number of limitations. The latter includes very limited use of participative design or other approaches to user involvement and limited end-user testing, which has often only involved blindfolded sighted people or a small number of participants. All the devices are still at the prototype stage, and there is a need to move beyond this and further develop and get devices to users. Finally, a number of suggested directions for future research have been presented.

## Figures and Tables

**Table 1:** Contributions and other features of the three other survey papers and this paper.

Contribution	Dakopoulos and Bourbakis 2009 [41]	Tapu et al., 2020 [42]	Velázquez 2010 [40]	This Paper
Types of devices surveyed	Wearable obstacle avoidance	Wearable and portable AT	Wearable AT	Wearable travel aids
Useful tables of device properties	x	x		x
Types of travel aid (ETA)	Obstacle avoidance			All
Parts of body travel aids worn on	Head, body, hand	Mainly head, some other	All	All
Types of sensors considered in ETAs	Mainly camera, some other	Mainly camera, some other	All	All
Types of user output in ETAs	All	All	Mainly tactile, some audio	All
Comparative evaluation	x	x		
Evaluation of device wearability and design				x
Classification of devices		x		x
Detailed recommendations for future work				x
Gaps in provision				x
Consider end-user testing	x	x		x
Consider end-user issues in design	x	x		x
Discuss device limitations				x
Design recommendations	x			x
Design and how/where ETAs worn				x

The ‘x’ indicates that the paper discusses the issue.

**Table 7:** Suggested improvements in form and wearability.

Reference	Smaller and Lighter	Different Size Options or Adjustable	Attached to User Choice of Glasses or Clothing
Abi Zeid Daou et al., 2020 [97]		x	x
Abu-Faraj et al., 2012 [98]		x	x
Agarwal et al., 2017 [59]	x		x
Alayon et al., 2020 [89]	x	x	
Anisha et al., 2021 [104]		x	x
Bai et al., 2019 [53]			
Balakrishnan et al., 2007 [46]	x		x
Bharathi et al., 2012 [62]	x		x
Bhatlawande et al., 2013 [86]		x	
Brilhault et al., 2011 [56]; Katz et al., 2012 [57]			x
Brock et al., 2014 [92]		x	
Caraiman et al., 2017 [51]			
Dakopoulos, 2009 [55]		x	
Diaz et al., 2020 [80]	x		x
Everding et al., 2016 [52]	x		x
Fiannaca et al., 2014 [50]			x
Fusiello et al., 2002 [44]			x
Gao et al., 2015 [71]		x	
Garcia-Macias et al., 2019 [73]			
Gay et al., 2020 [81]		x	x
Hsieh et al., 2020 [82]	x	x	
Huang et al., 2017 [93]	x		
Jameson and Manduchi 2010 [75]			
Khampachua et al., 2016 [88]	x	x	
Kuc, 2002 [87]		x	
Kumar et al., 2021 [100]		x	x
Laubhan et al., 2016 [61]	x		
Lee and Medioni, 2014 [54]	x		x
Leung et al., 2014 [58]			x
Li et al., 2016 [79]			
Li et al., 2017 [102]			
Lin et al., 2019 [49]	x		x
Linn et al., 2017 [85]			x
Mancini et al., 2018 [91]			x
Manikandan and Hussain, 2017 [105]		x	x
Mattoccia and Macri, 2014 [48]	x		x
Meijer, 1992 [47]; Dakopoulos and Bourbakis, 2009 [41]		x	x
Mocanu et al., 2016 [77]		x	
Molina et al., 2015 [74]			
Pradeep et al., 2010 [63]	x		x
Prathipa et al., 2019 [69]		x	
Riehle et al., 2013 [83]			
Sayed et al., 2020 [65]			x
Shoval et al., 1998 [68]		x	x
Tanveer et al., 2015 [60]			x
Tapu et al., 2013 [78]		x	
Tsukada and Yasumura 2004 [70]			x
Velazquez et al., 2006 [45]			x
Velazquez et al., 2018 [96]		x	x
Venkateswar and Mehendale, 2012 [67]			x
Villamizar et al., 2013 [72]			
Willis and Helal, 2005 [101]		x	x
Yang et al., 2018 [99]		x	x
Yeboah et al., 2018 [76]			x
Zelek et al., 2003 [90]			

The ‘x’ indicates improvements that might be beneficial.

**Table 8:** Suggested improvements in use and functionality.

Reference	Add High or Distant Obstacle Detection	Bone Conduction Headphones	Additional Languages	Optimise Number of Vibrators	Make Real Time	Improve Battery Life
Abi Zeid Daou et al., 2020 [97]		x	x			x
Abu-Faraj et al., 2012 [98]		x				
Agarwal et al., 2017 [59]		x				?
Alayon et al., 2020 [89]	x			x		?
Anisha et al., 2021 [104]		x				?
Bai et al., 2019 [53]		x	x		?	?
Balakrishnan et al., 2007 [46]		x			x	
Bharathi et al., 2012 [62]		x				x
Bhatlawande et al., 2013 [86]		x				x
Brilhault et al., 2011 [56]; Katz et al., 2012 [57]					?	
Brock et al., 2014 [92]						?
Caraiman et al., 2017 [51]		x			?	
Dakopoulos, 2009 [55]				x	?	
Diaz et al., 2020 [80]					?	x
Everding et al., 2016 [52]		x			?	
Fiannaca et al., 2014 [50]		x	x		?	
Fusiello et al., 2002 [44]		x			?	
Gao et al., 2015 [71]		x				
Garcia-Macias et al., 2019 [73]						
Gay et al., 2020 [81]				x	?	
Hsieh et al., 2020 [82]		x			?	
Huang et al., 2017 [93]						?
Jameson and Manduchi 2010 [75]		x				
Khampachua et al., 2016 [88]		x				?
Kuc, 2002 [87]						?
Kumar et al., 2021 [100]		x	x			?
Laubhan et al., 2016 [61]		x	x			
Lee and Medioni, 2014 [54]						
Leung et al., 2014 [58]					?	
Li et al., 2016 [79]		x	x		?	
Li et al., 2017 [102]	x					?
Lin et al., 2019 [49]		x	x			
Linn et al., 2017 [85]	x					x
Mancini et al., 2018 [91]					?	?
Manikandan and Hussain, 2017 [105]	x					?
Mattoccia and Macri, 2014 [48]	x				?	
Meijer, 1992 [47]; Dakopoulos and Bourbakis, 2009 [41]		x		x	?	
Mocanu et al., 2016 [77]			x			
Molina et al., 2015 [74]						
Pradeep et al., 2010 [63]					?	
Prathipa et al., 2019 [69]	x	x	x			
Riehle et al., 2013 [83]		x	x			
Sayed et al., 2020 [65]						
Shoval et al., 1998 [68]		x				
Tanveer et al., 2015 [60]	x	x				
Tapu et al., 2013 [78]						
Tsukada and Yasumura 2004 [70]				x		
Velazquez et al., 2006 [45]				x	?	
Velazquez et al., 2018 [96]						?
Venkateswar and Mehendale, 2012 [67]	x	x	x			
Villamizar et al., 2013 [72]						
Willis and Helal, 2005 [101]				x		?
Yang et al., 2018 [99]	x					?
Yeboah et al., 2018 [76]	x	x	x			x
Zelek et al., 2003 [90]				x	?	x

The ‘x’ indicates improvements that might be beneficial and the ‘?’ indicates a lack of information about whether the device already includes the potential improvement.

## Data Availability

Not applicable.
